# Supplementary material for: Modified Fluoroquinolones as Antimicrobial Compounds Targeting Chlamydia trachomatis
Source: Int J Mol Sci. 2022 Jun 16;23(12):6741. doi: 10.3390/ijms23126741 (PMC9224431; doi:10.3390/ijms23126741)
Supplement: Supplementary file 1 [file ijms-23-06741-s001.zip › ijms-1764518-supplementary.pdf]

Supporting Information

Modified fluoroquinolones as antimicrobial compounds targeting *Chlamydia trachomatis*.

Thi Huyen Vu,[1] Erika Adhel,[2] Katarina Vielfort,[3] Ngûyet-Thanh Ha-Duong,[2] Guillaume Anquetin,[2] Katy Jeannot,[4,5] Philippe Verbeke,[6] Sofia Hjalmar,[7] Åsa Gylfe, [7,*] and Nawal Serradji [2,*]

[1] University of Engineering and Technology, Vietnam National University, Hanoi (VNUH), Vietnam

[2] Université Paris Cité, CNRS, ITODYS, F-75013 Paris, France

[3] Department of Molecular Biology, Umeå University, SE-901 87 Umeå, Sweden

[4] Centre National de Référence de la Résistance aux Antibiotiques, Centre Hospitalier Universitaire de Besançon, Besançon, France

[5] Chrono-environnement UMR6249, CNRS Université de Faculté de Médecine-Pharmacie, Université Bourgogne-Franche Comté, F-25030, Besançon, France

[6] Université Paris Cité, INSERM U1149, Faculté de médecine Xavier Bichat, F-75018 Paris, France

[7] Department of Clinical Microbiology, Umeå University, SE-901 85 Umeå, Sweden

A. Organic synthesis
B. Iron chelating properties of compounds **5a-d** at pH 2.
C. Iron chelating properties of compound **6** at pH 2.
D. Antichlamydial activity of the antimicrobials.
E. ^{1}H and ^{13}C NMR of the final compounds

A. Organic synthesis

General procedure of alkylation of 2-methylquinolin-8-ol (compounds 2b-d)

Alkyl halide (1.5 eq.) was added to a mixture of 2-methylquinolin-8-ol and K_2CO_3 (1.5 eq.) in DMF (10 mL) and under argon. After reaction completion (TLC), DMF was removed under vacuum. Water (30 mL) was added to the crude compound and the aqueous layer was extracted twice with dichloromethane (80 mL). The combined organic layers were dried over anhydrous $MgSO_4$, filtered and concentrated under reduced pressure. The residue was purified by silica gel column chromatography to provide compounds **2b-d** as solids.

Synthesis of 2-Methyl-8-propylquinoline (2b)

From **p**ropyl bromide (703.30 μL, 9.42 mmol) and following the general procedure of alkylation. The residue was purified by silica gel column chromatography (elution: CH_2Cl_2) to obtain compound **2b (**1.174 g, 99%) as solid. IR (KBr, ν cm^{-1}): 2976, 2923 (CH), 1603 ($C{=}C_{ar}$). ^1H NMR ($CDCl_3$) δ ppm: 8.00 (d, *J*=8.40 Hz, 1H), 7.28–7.40 (m, 3H), 7.04 (d, *J*=7.20 Hz, 1H), 4.35 (q, *J*=7.20 Hz, 2H), 2.80 (s, 3H), 1.63 (t, *J*=6.80 Hz, 3H). ^{13}C NMR ($CDCl_3$) δ ppm: 157.8, 154.4, 140.3, 135.8, 127.7, 125.5, 122.1, 119.3, 109.5, 64.6, 25.4, 14.4.

8-Butoxy-2-méthylquinoline (2c)

From **b**utyl bromide (1.2 g, 9.42 mmol) and following the general procedure for alkylation. The crude product was purified using silica gel chromatography with the mixture of MeOH/CH_2Cl_2 (0.5:99.5, v/v) as eluent to provide compound **2c** (1.93 g, 95%) as solid. IR (KBr, ν cm^{-1}): 2956 (CH_{ar}), 2869 (OCH_3), 1602 ($C{=}C_{ar}$). ^1H NMR ($CDCl_3$) δ ppm: 1.02 (t, *J* =7.40 Hz, 3H), 1.56 (sext, *J* =7.50 Hz, 2H), 2.00 (qt, *J* =7.40 Hz, 2H), 2.76 (s, 3H), 4.22 (t, 2H, *J* =7.20 Hz), 7.03 (d, *J* =7.50 Hz, 1H), 7.35-7.25 (m, 3H), 7.98 (d, *J* =8,10

Hz, 1H). ^{13}C NMR ($CDCl_3$) δ ppm: 13.9, 19.4, 25.7, 31.3, 69.4, 109.9, 119.5, 122.4, 125.7, 128.0, 136.0, 154.9, 158.0.

8-(Benzyloxy)-2-methylquinoline (**2d**)

From **b**enzyl bromide (1.12 mL, 9.42 mmol) and KI (104.3 mg, 0.6282 mmol) in refluxing acetone (10 mL) and following the general procedure for alkylation. The residue was crystallized in hexane to provide compound **2d** (1.39 g, 88%) as solid. IR (KBr, ν cm^{-1}): 2932, 2878 (CH), 1604 ($C{=}C_{ar}$). ^{1}H NMR ($CDCl_3$) δ ppm: 8.0 (d, *J*=8.40 Hz, 1H), 7.52 (m, 1H), 7.26-7.39 (m, 7H), 7.02 (dd, *J*=7.20 Hz and 1.20 Hz, 1H), 5.46 (s, 2H), 2.81 (s, 3H). ^{13}C NMR ($CDCl_3$) δ ppm: 158.2, 154.4, 140.7, 137.8, 136.1, 128.7, 128.0, 127.7, 127.6, 125.6, 122.5, 120.2, 111.5, 71.6, 25.8.

Oxidation using selenium dioxide – general procedure (compounds 3b-d)

2-methyl-8-alkylquinoline **2b-d were** reacted with SeO_2 (1.1 eq.) in refluxing and degassed 1,4-dioxane (10 mL) for 2.5 hours. The mixture was then cooled to room temperature. The precipitate was filtered and washed several times using dichloromethane. The combined filtrates were concentrated under vacuum and the residue was purified as described below to provide compounds **3b-d**.

8-Propylquinoline-2-carbaldehyde (3b)

From 2-methyl-8-propyloxyquinoline (1.164 g, 6.220 mmol) and following the general procedure using selenium dioxide. A silica gel column chromatography (solvent: $MeOH/CH_2Cl_2$, 1:99, v/v) provided compound **3b** (0.378 g, 31%) as oil. IR (KBr, ν cm^{-1}): 2830, 2935, 2980 (CH), 1707 (CHO). ^{1}H NMR ($CDCl_3$) δ ppm: 10.30 (s, 1H), 8.27 (d, *J*=8.40 Hz, 1H), 8.06 (d, *J*=8.40 Hz, 1H), 7.60 (t, *J*=8.0 Hz, 1H), 7.46 (d, *J*=8.00 Hz, 1H), 7.16 (d, *J* = 8.00 Hz, 1H), 4.40 (q, *J*=7.20 Hz, 2H), 2.72 (t, *J*=6.80 Hz, 3H). ^{13}C NMR ($CDCl_3$) δ ppm: 193.5, 151.6, 140.4, 137.0, 131.4, 129.5, 119.4, 117.6, 110.0, 65.0, 14.5.

8-Butoxyquinoline-2-carbaldehyde (3c)

From 8-butyloxy-2-methylquinoline (665 mg, 6.28 mmol) and following the general procedure using selenium dioxide. The crude compound **3c** was used in the next step without purification. IR (KBr, ν cm^{-1}): 2958 (CH_{ar}), 2873 (OCH_2), 1707 (C=O). ^1H NMR ($CDCl_3$) δ ppm: 10.27 (s, 1H), 8.25 (d, *J* =8.40 Hz, 1H), 8.03 (d, *J* =8.40 Hz, 1H), 7.58 (t, *J*=8.00 Hz, 1H), 7.43 (d, *J* = 8.20 Hz, 1H), 7.13 (d, *J* =7.70 Hz, 1H), 4.29 (t, *J* =6.90 Hz, 2H), 2.04 (quint, *J* =7.10 Hz, 2H), 1.60 (sext, *J* =7.50 Hz, 2H), 1.05 (t, *J* =7.30 Hz, 3H). ^{13}C NMR ($CDCl_3$) δ ppm: 193.8, 156.2, 151.8, 137.2, 131.6, 129.82, 119.6, 117.8, 110.4, 69.6, 31.3, 19.4, 13.9.

8-(Benzyloxy)quinolone-2-carbaldehyde (**3d**)

From 8-(benzyloxy)-2-methylquinoline (500 mg, 2 mmol) and following the general procedure using selenium dioxide. The mixture was then heated at 80 °C under argon for 3 hours (no more starting material could be detected; TLC: DCM/MeOH 98:2). The residue was purified using silica gel column chromatoghraphy (solvent: CH_2Cl_2) to obtain compound **3d** (352.7 mg, 67%) as yellow-brown solid. IR (KBr, ν cm^{-1}): 3068-2836 (CH), 1696 (C=O), 1657 (C=N), 1614 ($C=C_{ar}$). ^1H NMR ($CDCl_3$) δ ppm: 10.32 (s, 1H), 8.28 (d, *J*=8.50 Hz, 1H), 8.08 (d, *J*=8.40 Hz, 1H), 7.54 (m, 3H), 7.47 (dd, *J*=8.20 Hz and 1.10 Hz,1H), 7.38 – 7.42 (m, 2H), 7.31 – 7.35 (m, 1H), 7.16 (dd, *J*=7.70 Hz and 1.1 Hz, 1H), 5.49 (s, 2H). ^{13}C NMR ($CDCl_3$) δ ppm: 193.9, 152.1, 137.4, 137.1, 131.8, 129.8, 128.9, 128.2, 127.5, 120.4, 118.0, 112.0, 71.9.

General procedure for oxidation using Oxone® (compounds 4b-c)

Oxone® (1.06 eq.) was added to a solution of aldehydes **3b-c** in DMF. The mixture was stirred at RT for 2 hours. The DMF was then removed under vacuum. The obtained residue was treated with 1N HCl (2 mL) to dissolve the salt. Then, ethyl acetate (15 mL) was added to extract the products. The combined organic extracts were washed

with water (2 mL) and brine (2 mL), dried over anhydrous $MgSO_4$ and filtered. After evaporation of the solvent, the crude product obtained was purified by silica gel column chromatography (elution: EtOAc/Hexane, 1:1, v/v) to provide compounds **4b-c**.

8-Propylquinoline-2-carboxylic acid (4b)

From 8-propyloxyquinoline-2-carbaldehyde **3b** (128 mg, 0.636 mmol) and following the general procedure using Oxone® in DMF (5 mL). A silica gel column chromatography (elution: EtOAc/Hexane, 1:1, v/v) provided compound **4b** (72 mg, 52%). TLC (1% MeOH/DCM) was used to monitor during reacting and purification. IR (KBr, ν cm^{-1}): 3185 (OH), 2942, 2985 (CH), 1720 (COOH). ^1H NMR ($CDCl_3$) δ ppm: 8.36 (d, *J*=8.4 Hz, 1H), 8.28 (d, *J*=8.40 Hz, 1H), 7.60 (t, *J*=8.00 Hz, 1H), 7.48 (d, *J*=8.00 Hz, 1H), 7.14 (d, *J*=7.60 Hz,1H), 4.30 (q, *J*=6.80 Hz, 2H), 1.62 (t, *J*=7.20 Hz, 3H). ^{13}C NMR ($CDCl_3$) δ ppm: 164.0, 155.0, 144.7, 138.3, 138.2, 131.2, 129.5, 119.4, 119.1, 110.2, 64.7, 14.5.

8-Butoxyquinoline-2-carboxylic acid (4c)

A solution of aldehyde **3c** (100 mg, 0.436 mmol) and Oxone (134.01 mg, 0.436 mmol) in DMF was stirred under argon for 16 h. After evaporation of DMF under vacuum, the residue obtained was dissolved in a 1N HCl solution and the organic phase was extracted with ethyl acetate and then washed with water, dried over $MgSO_4$, evaporated and purified on silica gel column using a mixture of MeOH/CH_2Cl_2 (5:95, v/v) as eluent to afford compound **4c** (68.1 mg, 63%). IR (KBr, ν cm^{-1}): 3434 (OH), 2957 (CH_{ar}), 2871 (OCH_2), 1641 (C=O), 1603 (C=C_{ar}). ^1H NMR ($CDCl_3$) δ ppm: 8.35 (d, *J* = 8.40 Hz, 1H), 8.26 (d, *J* = 8.40 Hz, 1H), 7.58 (t, *J* = 8.10 Hz, 1H), 7.48 (d, 1H, *J* = 8.30 Hz, 1H), 7.13 (d, *J* =7.70 Hz, 1H), 4.22 (t, *J* = 6.50 Hz, 2H), 1.95 (qt, *J* = 7.10 Hz, 2H), 1.58 (sext, *J* = 7.50 Hz, 2H), 1.03 (t, *J* = 7.30 Hz, 3H). ^{13}C NMR ($CDCl_3$) δ ppm:

164.3, 155.4, 144.9, 138.6, 138.5, 131.5, 129.8, 119.6, 119.3, 110.5, 69.3, 31.4, 19.6, 13.9.

8-(Benzyloxy)quinolone-2-carboxylic acid (**4d**)

H_2NSO_3H (221.3 mg, 2.28 mmol) and $NaClO_2$ (206.1 mg, 2.28 mmol) were added to a cooled solution of 8-(benzyloxy)quinolone-2-carbaldehyde **3d** (150 mg, 0.57 mmol) in THF (4 mL) and H_2O (3 mL). After one hour of stirring, water (25 mL) was slowly added under sonication for 45 minutes to provide a white precipitate which was filtered, washed with water, and dried under vacuum to provide compound **4d** (144.6 mg, 91%). IR (KBr, ν cm^{-1}): 3490, 3360 (OH), 3065, 2918 (CH), 1714 (C=O), 1504 ($C=C_{ar}$). ^1H NMR ($CDCl_3$) δ ppm: 8.37 (d, *J*=8.40 Hz, 1H), 8.29 (d, *J*=8.40 Hz, 1H), 7.59 (t, J=8.00 Hz, 1H), 7.52 (m, 3H), 7.35-7.45 (m, 3H), 7.21 (d, J=7.60 Hz, 1H), 5.35 (s, 2H). ^{13}C NMR ($CDCl_3$) δ ppm: 164.3, 155.1, 138.7, 136.7, 131.5, 129.7, 128.9, 128.4, 127.4, 120.0, 119.8, 111.7, 71.6.

General procedure A for ciprofloxacin (Cip) coupling (compounds 5a-d, 6)

TBTU (1 eq.) and DIEA (1.2 eq.) were added to a solution of carboxylic acid (1 eq.) in anhydrous DMF (7 mL). After 15 min of stirring under argon, ciprofloxacin (0.9 eq.) was added and the resulting suspension was stirred for 3 days. The solvent was then removed under vacuum. The residue was then purified as described below to provide compounds **5a-d** and **6**.

7-(4-(8-(Methyloxy)quinoline-7-carbonyl)piperazin-1-yl)-1-cyclopropyl-6-fluoro-4-oxo-1,4-dihydroquinoline-3-carboxylic acid (5a)

From **commercially available** 8-methoxyquinoline-2-carboxylic acid (50mg, 0.248 mmol) and following general procedure **A** for Cip coupling. Trituration in MeOH provided compound **5a** (92 mg, 71%) as yellow solid. Mp > 260°C. IR (KBr, ν cm^{-1}): 3471 (OH), 1712, 1625 (C=O). ^1H NMR (DMSO-d_6) δ ppm: 15.07 (s, 1H), 8.67 (s, 1H), 8.38 (d, *J*=8.48 Hz, 1H), 7.91 (d, *J*=13.04 Hz, 1H), 7.74 (d, *J*=8.44 Hz, 1H), 7.50-7.57 (m, 3H), 7.19 (d, *J*=7.36 Hz, 1H), 3.98 (m, 5H), 3.87 (m, 2H), 3.77 (m, 1H), 3.51 (m, 2H), 3.38 (m, 2H), 1.31 (m, 2H), 1.18 (m, 2H). ^{13}C NMR (DMSO-d_6) δ ppm: 176.1, 166.5, 165.7,

155.1, 151.6, 147.6, 144.5 (d, *J*=9.27 Hz), 138.7, 136.9, 128.7, 127.8, 120.8, 119.1, 111.2, 110.9 (d, *J*=23.03 Hz), 108.7, 106.9, 106.2, 55.7, 49.6, 49.1, 46.3, 41.4, 35.5, 7.5. HRMS *m*/*z* calcd for $C_{28}H_{26}FN_4O_5$ $[M+H]^+$ 517.1887; found 517.1894.

7-(4-(8-(Propyloxy)quinoline-7-carbonyl)piperazin-1-yl)-1-cyclopropyl-6-fluoro-4-oxo-1,4-dihydroquinoline-3-carboxylic acid (5b)

From 8-propyloxyquinoline-2-carboxylic acid **4b** (51 mg, 0.235 mmol) and following general procedure **A** for Cip coupling. Trituration in MeOH provided compound **5b** (97 mg, 78%) obtained as solid. Mp > 260°C. IR (KBr, ν cm^{-1}): 3458 (OH), 3041, 2895 (CH), 1719, 1619 (C=O). ^1H NMR ($CDCl_3$) δ ppm: 14.93 (s, 1H), 8.79 (s, 1H), 8.26 (d, *J*=8.40 Hz, 1H), 8.07 (d, *J*=13.00 Hz, 1H), 7.95 (d, *J*=8.5 Hz, 1H), 7.53 (d, *J*=8.0 Hz, 1H), 7.43 (m, 2H), 7.10 (d, *J*=7.60 Hz, 1H), 4.27 (m, 4H), 4.12 (m, 2H), 3.49-3.56 (m, 4H), 1.56 (t, *J*=6.80 Hz, 3H), 1.40 (m, 2H), 1.21 (m, 2H). ^{13}C NMR ($CDCl_3$) δ ppm: 176.1, 166.5, 165.7, 155.1, 151.6, 147.4, 139.1, 136.8, 128.2, 122.0, 119.3, 104.8, 64.6, 35.1, 14.7, 8.1. HRMS *m*/*z* calcd for $C_{29}H_{28}FN_4O_5$ $[M+H]^+$ 531.2044; found 531.2034.

7-(4-(8-Butoxyquinoline-2-carbonyl)piperazin-1-yl)-1-cyclopropyl-6-fluoro-8-méthoxy-4-oxo-1,4-dihydroquinoline-3-carboxylic acid (5c)

From 8-butyloxyquinoline-2-carboxylic acid **4c** (106.7 mg, 0.435 mmol) and following the general procedure **A** for Cip coupling. Trituration in MeOH provided compound **5c** (143 mg, 77%) obtained as solid. Mp > 260°C. IR (KBr, ν cm^{-1}): 2918 (OH), 1626 (C=O). ^1H NMR (DMSO-d_6) δ ppm: 8.47 (s, 1H), 8.45 (d, *J* = 8.56 Hz, 1H), 7.80 (m, 2H), 7.55 (m, 2H), 7.45 (d, *J* = 7.36 Hz, 1H), 7.23 (d, *J* = 7.28 Hz, 1H), 4.16 (t, *J* = 6.63 Hz, 2H), 3.94 (m, 4H), 3.56 (m, 1H), 3.37 (m, 4H), 1.77 (qt, *J* = 6.84 Hz, 2H), 1.49 (sext, *J* =7.44 Hz , 2H), 1.26 (m, 2H), 0.99 (m, 2H), 0,84 (t, *J* = 7.36 Hz, 3H). ^{13}C NMR (DMSO-d_6) δ ppm: 166.1, 146.4, 136.7, 128.6, 127.8, 121.2, 120.8, 119.3, 118.9, 111.6, 109.9, 105.8, 68.1, 50.2, 49.3, 46.4, 41.5, 33.7, 30.4, 18.4, 13.1, 7.1. HRMS *m*/*z* calcd for $C_{31}H_{32}FN_4O_5$ $[M+H]^+$ 559.2357; found 559.2366.

7-(4-(8-(Benzyloxy)quinoline-7-carbonyl)piperazin-1-yl)-1-cyclopropyl-6-fluoro-4-oxo-1,4-dihydroquinoline-3-carboxylic acid (5d)

From 8-(benzyloxy)quinoline-2-carboxylic acid **4d** (50 mg, 0.179 mmol) and following the general procedure **A** for Cip coupling. Trituration in MeOH provided compound **5d** (75.5 mg, 71%) obtained as solid. Mp > 260°C. IR (KBr, ν cm^{-1}): 3448 (OH), 3021-2865 (CH), 1726, 1616 (C=O). ^1H NMR (CD_2Cl_2) δ ppm: 14.97 (s, 1H), 8.78 (s, 1H), 8.32 (d, *J*=8.40 Hz, 1H), 8.06 (d, *J*=12.80 Hz, 1H), 7.88 (d, *J*=8.40 Hz, 1H), 7.49-7.58 (m, 4H), 7.41 (d, *J*=6.80 Hz, 1H), 7.29 (m, 2H), 7.14–7.21 (m, 2H), 5.28 (s, 2H), 4.15 (m, 2H), 4.09 (m, 2H), 3.47 (m, 3H), 3.41 (m, 2H), 1.32 (m, 2H), 1.17 (m, 2H). ^{13}C NMR (CD_2Cl_2) δ ppm: 167.6, 148.2, 137.5, 130.4, 129.1, 128.7, 128.6, 128.1, 122.4, 120.6, 106.1, 35.8, 8.7. HRMS *m*/*z* calcd for $C_{34}H_{30}FN_4O_5$ $[M+H]^+$ 593.2221; found 593.2200.

1-Cyclopropyl-6-fluoro-7-(4-(8-hydroxyquinoline-7-carbonyl)piperazin-1-yl)-4-oxo-1,4-dihydroquinoline-3-carboxylic acid (6)

From **commercially available** 8-hydroxyquinoline-7-carboxylic acid (30 mg, 0.158 mmol) and following the general procedure **A** for Cip coupling (the mixture was heated at 80°C for 24 hrs). Trituration in MeOH provided compound **6** as solid (30 mg, 37%). Mp > 260°C. IR (KBr, ν cm^{-1}): 3400, 3304 (OH), 1732, 1626 (C=O). ^1H NMR (DMSO-d_6) δ ppm: 15.19 (s, 1H), 8.93 (d, *J* = 2.96 Hz, 1H), 8.67 (s, 1H), 8.39 (dd, *J*=8.00 Hz and 1.20 Hz, 1H), 7.93 (d, *J*=13.20 Hz, 1H), 7.59 – 7.66 (m, 2H), 7.49 (d, *J*=8.80 Hz, 1H), 7.43 (d, *J*=8.40 Hz, 1H), 3.93 (m, 2H), 3.82 (m, 2H), 3.61 (m, 1H), 3.51 (m, 2H), 3.45 (m, 2H), 1.32 (m, 2H), 1.18 (m, 2H). ^{13}C NMR (DMSO-d_6) δ ppm: 175.9, 166.6, 165.2, 148.7, 148.5, 147.5, 144.8 (d, *J*=10.25 Hz), 138.9, 135.8, 126.0, 122.3, 118.7, 117.7, 111.0 (d, *J*=22.65 Hz), 49.2, 35.4, 7.2. HRMS *m*/*z* calcd for $C_{27}H_{24}FN_4O_5$ $[M+H]^+$ 503.1731; found 503.1706.

General procedure B for ciprofloxacin coupling (indole series 7-12)

TBTU (1.11 eq.) and DIEA (1.33 eq.) were added to a solution of carboxylic acid (1.11 eq.) in anhydrous DMF (7 mL). After 15 min of stirring under argon, ciprofloxacin (1 eq.) was added and the resulting suspension was stirred for 2-4 days. The solvent was then removed under vacuum. The residue was purified as described below to provide compounds **7-12**.

7-(4-(1*H*-Indole-2-carbonyl)piperazin-1-yl)-1-cyclopropyl-6-fluoro-4-oxo-1,4-dihydroquinoline-3-carboxylic acid (**7**)

From indole-2-carboxylic acid (0.0425 g, 0.264 mmol) and following the general procedure **B** for Cip coupling, the final compound **7** (93 mg, 82%) was obtained after filtration under vacuum as white solid. Mp > 260°C. IR (KBr, ν cm^{-1}): 3432 (NH), 3268 (COOH), 3062 (Ar), 1719 (C=O), 1504 (ArC=C). ^{1}H NMR (DMSO-d_6) δ ppm**:** 15.2 (s, 1H), 11.6 (s, 1H), 8.7 (s, 1H), 7.9 (m, 1H), 7.6 (m, 2H), 7.4 (d, *J*=8.16 Hz, 1H), 7.2 (m, 1H), 7.06 (t, *J*= 7.40 Hz, 1H), 6.9 (s, 1H), 4 (br s, 4H), 3.8 (m, 1H), 3.4 (br s, 4H), 1.3 (m, 2H), 1.2 (m, 2H). ^{13}C NMR (DMSO-d_6) δ ppm: 176.3, 165.5, 162.0, 147.6, 139.0, 135.8, 129.5, 126.7, 123.0, 121.0, 119.5, 111.8 (d, *J*= 23,14 Hz), 110.9, 110.7, 106.1, 104.0, 54.5, 49.2, 35.5, 7.3. HRMS *m*/*z* calcd for $C_{26}H_{24}FN_4O_4$ $[M+H]^+$ 475.1782; found 475.1798.

7-(4-(1*H*-Indole-3-carbonyl)piperazin-1-yl)-1-cyclopropyl-6-fluoro-4-oxo-1,4-dihydroquinoline-3-carboxylic acid (8)

From indole-3-carboxylic acid (0.0425 g, 0.264 mmol) and following the general procedure **B** for Cip coupling. A trituration in MeOH led to compound **8** (67.2 mg, 68%) as white solid. Mp > 260°C. IR (KBr, ν cm^{-1}): 3419 (NH), 3050 (Ar), 1725 (C=O), 1536 (ArC=C), 1513 (NH). ^{1}H NMR (DMSO-d_6) δ ppm: 15.2 (br s, 1H), 11.6 (s, 1H), 8.7 (s, 1H), 7.9 (d, *J* = 13.04 Hz, 1H); 7.8 (m, 2H) , 7.6 (d, *J* = 7.40 Hz, 1H) ; 7.4 (d, *J* = 7.96 Hz, 1H), 7.1 (m, 2H), 3.9 (m, 4H), 3.8 (m, 1H), 3.4 (m, 4H), 1.3 (m, 2H), 1.2 (m, 2H). ^{13}C NMR (DMSO-d_6) δ ppm: 176.2, 165.5, 147.6, 145.0, 139.1, 135.8, 125.9, 121.6, 119.9, 118.7,

111.6, 110.8 (d, *J*= 27.2 Hz), 49.4, 44.0, 35.5, 7.3. HRMS *m/z* calcd for $C_{26}H_{24}FN_4O_4$ $[M+H]^+$ 475.1782; found 475.1773.

1-Cyclopropyl-6-fluoro-7-(4-(5-hydroxy-1*H*-indole-2-carbonyl)piperazin-1-yl)-4-oxo-1,4-dihydroquinoline-3-carboxylic acid (9)

From 5-hydroxyindole-2-carboxylic acid (0.0352 g, 0.264 mmol) and following the general procedure **B** for Cip coupling. A filtration under vacuum led to compound **9** (111 mg, 95%) as white solid. Mp > 260°C. IR (KBr, ν cm^{-1}): 3149 (OH), 3083 (ArC=C), 1705 (C=O), 1532 (ArC=C), 1508 (NH). ^{1}H NMR (DMSO-d_6) δ ppm: 15.2 (s, 1H), 11.3 (s, 1H), 8.8 (s, 1H), 8.6 (s, 1H), 7.9 (m, 1H), 7.61 (d, *J* = 7.40 Jz, 1H), 7.25 (d, *J* = 8.76 Hz, 1H), 6.9 (m, 1H), 6.74 (dd, *J* = 8.76 and 2.24 , 1H), 6.71 (m, 1H), 3.9 (m, 4H), 3.8 (m, 1H), 3.4 (m, 4H), 1.4 (m, 2H), 1.2 (m, 2H). ^{13}C NMR (DMSO-d_6) δ ppm: 176.1, 165.6, 150 .9, 157.6, 144.5 (d, *J*=10.40 Hz), 130.6, 129.6, 127.4, 114.3, 112.2, 110.7 (d, *J*=26.72 Hz), 106.1, 104.1, 103.2, 49.2, 43.9, 35.5, 30.5, 7.3. HRMS *m/z* calcd for $C_{26}H_{24}FN_4O_5$ $[M+H]^+$ 491.1731; found 491.1732.

7-(4-(2-(1*H*-Indol-3-yl)-2-oxoacetyl)piperazin-1-yl)-1-cyclopropyl-6-fluoro-4-oxo-1,4-dihydroquinoline-3-carboxylic acid (10)

A suspension of indole (0.1000 g, 0.853 mmol) in Et_2O (5 mL) was stirred in an ice bath for 10 min. Oxalyl chloride (88 μL, 1.02 mmol) was then added dropwise to the cold mixture. After 1 hr of stirring, the yellow solid was filtered under vacuum to afford 2-(1H-Indol-3-yl)-2-oxoacetyl chloride (37 mg, 37%). IR (KBr, ν cm^{-1}): 3441 (NH), 3056 (ArC=C), 1783 (C=O). ^{1}H NMR (DMSO-d_6) δ ppm: 12.4 (s, 1H), 8.4 (d, *J* = 3.24 Hz, 1H), 8.16 (dd, *J* = 6.88 and 2.20 Hz, 1H), 7.53 (m, 1H), 7.26 (m, 2H). ^{13}C NMR (DMSO-d_6) δ ppm: 180.4, 164.8, 137.2, 136.4, 125.4, 123.3, 122.3, 120.8, 112.3, 112.2.

2-(1H-Indol-3-yl)-2-oxoacetyl chloride (0.0450 g, 0.241 mmol) was then added portion wise to a suspension of ciprofloxacin (0.0799 g, 0.241 mmol) in DMF (5 mL). DIEA (54.4 μL, 0.313 mmol) was then added to the mixture which was stirred at RT for 1 hr. The

solvent was removed under vacuum and the residue triturated in MeOH to provide **10** (53.6 mg, 44%) as solid. Mp = 230±2°C. IR (KBr, ν cm^{-1}): 3468 (NH), 3217 (COOH), 3066 (ArC=C), 1722 (C=O). ^{1}H NMR (DMSO-d_6) δ ppm**:** 15.1 (s, 1H), 12.3 (s, 1H), 8.7 (s, 1H), 8.24 (m, 1H), 8.14 (m, 1H), 7.93 (d, *J* = 6.40 Hz, 1H), 7.56 (m, 2H), 7.28 (m, 2H), 3.86 (m, 2H), 3.6 (m, 1H), 3.59 (m, 2H), 3.49 (m, 2H), 1.3 (m, 2H), 1.2 (m, 2H). ^{13}C NMR (DMSO-d_6) δ ppm: 185.5, 176.15, 165.7, 165.4, 147.6, 144.3 (d, *J*=10.32 Hz), 138.9, 136.7, 124.7, 123.3, 122.3, 120.6, 113.0, 112.3, 110.8 (d, *J*=24.93 Hz), 106.8, 106.4, 49.3, 48.9, 44.9, 35.5, 7.3. HRMS *m/z* calcd for $C_{27}H_{24}FN_4O_4$ $[M+H]^+$ 503.1731; found 503.1747.

7-(4-(2-(1*H*-Indol-3-yl)acetyl)piperazin-1-yl)-1-cyclopropyl-6-fluoro-4-oxo-1,4-dihydroquinoline-3-carboxylic acid (11)

From indole-3-acetic acid (0.0431 g, 0.264 mmol) and following the general procedure **B** for Cip coupling. A trituration in MeOH led to **11** (71%) as white solid. Mp > 260°C. IR (KBr, ν cm^{-1}): 3418 (NH), 3272 (COOH), 3055 (ArC=C), 1725 (C=O). ^{1}H NMR (DMSO-d_6) δ ppm**:** 15.2 (s, 1H), 10.9 (s, 1H), 8.65 (s, 1H), 7.91 (d, *J* = 13.20 Hz, 1H), 7.56 (d, *J* = 7.81 Hz, 1H), 7.50 (d, *J* = 7.40 Hz, 1H), 7.34 (d, *J* = 8.08 Hz, 1H), 7.24 (m, 1H), 7.07 (t, *J* = 7.12 Hz, 1H), 7.0 (t, *J* = 7.64 Hz, 1H), 3.9 (s, 1H), 3.84 (s, 2H), 3.76 (m, 2H), 3.71 (m, 2H), 3.27 (m, 2H), 3.21 (m, 2H), 1.3 (m, 2H), 1.2 (m, 2H). ^{13}C NMR (DMSO-d_6) δ ppm: 185.5, 176.15, 165.7, 165.4, 147.6, 144.5 (d, *J*=9.22 Hz), 138.9, 136.7, 124.7, 123.3, 122.3, 120.6, 113.0, 112.3, 110.7 (d, *J*=23.57 Hz), 106.8, 106.4, 49.3, 48.9, 44.9, 35.5, 7.3. HRMS *m/z* calcd for $C_{27}H_{26}FN_4O_4$ $[M+H]^+$ 489.1938; found 489.1954.

7-(4-(5-Chloro-1*H*-indole-2-carbonyl)piperazin-1-yl)-1-cyclopropyl-6-fluoro-4-oxo-1,4-dihydroquinoline-3-carboxylic acid (12)

From 5-chloroindole-2-carboxylic acid (0.0516 g, 0.264 mmol) and following the general procedure **B** for Cip coupling. A trituration in MeOH led to **12** (117 mg, 97%) as white solid. IR (KBr, ν cm^{-1}): 3432 (NH), 3257 (COOH), 3070 (ArC=C), 1726 (C=O), 1511 (NH). ^{1}H NMR (DMSO-d_6) δ ppm**:** 15.2 (s, 1H), 11.86 (s, 1H), 8.67 (s, 1H), 7.94 (m, 2H), 7.67 (m, 1H), 7.61 (d, *J* = 7.40 Hz, 1H), 7.44 (d, *J* = 8.72 Hz, 1H), 7.2 (dd, *J* = 8.68 and

2.00 Hz, 1H), 6.88 (s, 1H), 4,0 (m, 4H), 3.81 (m, 1H), 3.46 (m, 4H), 1.3 (m, 2H), 1.2 (m, 2H). ^{13}C NMR (DMSO-d_6) δ ppm: 205.7, 176.1, 165.4, 161.6, 147.6, 144.4 (d, *J*=11.02 Hz), 138.9, 134.2, 131.1, 127.7, 123.1, 120.0, 113.4, 111.0, 110.8 (d, *J*=23.31 Hz), 106.8, 106.0, 103.4, 49.1, 35.5, 35.4, 30.2, 7.3. HRMS *m/z* calcd for $C_{26}H_{23}ClFN_4O_4$ $[M+H]^+$ 509.1392; found 509.1411.

1-Cyclopropyl-6-fluoro-7-(4-((8-hydroxyquinoline-5-yl)méthyl)pipérazin-1-yl)-4-oxo-1,4-dihydroquinoline-3-carboxylic acid (14) :

A mixture of ciprofloxacin (0.100 g, 0.277 mmol), 5-chloromethyl-8-hydroxyquinoline, hydrochloride **13** [23] (0.00584 g, 0,332 mmol), K_2CO_3 (0.0776 g, 0,554 mmol) in DMF (7 mL) was stirred at RT for 24 hrs. The resulting suspension was then filtered under vacuum. The residue was first recrystallized in hot MeOH and chromatographed on a silica gel column, using MeOH /CH_2Cl_2, 5:95, v/v) as eluent, to afford compound **14** (55 mg, 84%). IR (KBr, ν cm^{-1}): 3432 (OH), 2932 (OH), 1721 (C=N), 1630 (C=O), 1565 (C=C arm). ^{1}H NMR (DMSO-d_6) δ ppm**:** 15.21 (s, 1H), 9.73 (s, 1H), 8.85 (d, 1H, *J* =4.08 Hz), 8.71(d, 1H, *J* =8.56 Hz), 8.64 (s, 1H), 7.89 (d, 1H, *J* =13.24), 7.60 (dd, 1H, *J*=4.12 and 8.56 Hz), 7.53 (d, 1H, *J* =7.48 Hz), 7.37 (d, 1H, *J* =7.76 Hz), 7.01 (d, 1H, *J* =7.72 Hz), 3.88 (s, 2H), 3.77 (m, 1H), 3.27 (m, 4H), 2.63 (m, 4H), 1.27 (m, 2H), 1.15 (m, 2H). ^{13}C NMR (DMSO-d_6) δ ppm: 181.4, 49.0, 152, 147.52, 147.4, 144.1 (d, *J*=9.68 Hz), 138.9, 133.3, 130.3, 128.5, 127.5, 121.1, 111.7 (d, *J*=22.86 Hz), 109.4, 106.0, 59.9, 59.5, 50.8, 49.2, 39.5, 35.4, 7.2. HRMS *m/z* calcd for $C_{27}H_{26}FN_4O_4$ $[M+H]^+$ 489.1938; found 489.1926.

1-cyclopropyl-6-fluoro-8-methoxy-4-oxo-7-(piperazin-1-yl)-1,4-dihydroquinoline-3-carboxylic acid (15):

(1-Cyclopropyl-6,7-difluoro-1,4-dihydro-8-methoxy-4-oxo-3-quinoline-carboxylato-O3-O4)difluoro-boron [1] (0.089 g, 0.26 mmol) was added to a suspension of piperazine (0.029 g, 0.338 mmol) in acetonitrile (5 mL). The mixture was stirred stirred under argon at RT for 5 min. Triethylamine (0.158 g, 1.56 mmol) was then added portionwise and the mixture was refluxed for 24 hr. The solution was cooled to 60 °C and ethanol

(2.1 mL) and triethylamine (0.158 g, 1.56 mmol) were added. The mixture was refluxed for 2 hr at 72 °C and then cooled at RT for 20 hr. The solvent was removed under vacuum and the residue triturated in cyclohexane to provide **15** (0.0641 g, 72%) as solid. Mp = 230±2°C. IR (KBr, ν cm^{-1}): 3380 (OH/NH), 2980 and 2900 (CH_3), 1670 (C=O), 1630 (ArC=C), 1570 (ArC=C), 1490 (ArC=C), 1119 (NR_3). ^1H NMR (DMSO-d_6) δ ppm: 8.69 (s, 1H), 7.74 (d, *J* = 12.3 Hz, 1H), 4.27 – 4.08 (m, 1H), 3.77 (s, 3H), 3.29-3.24 (m, 4H), 2.89-2,83 (m, 4H), 1.40 (s, 1H), 1.23 – 0.87 (m, 4H). ^{13}C NMR (DMSO-d_6) δ ppm: 176.8, 166.2, 157.3, 154.8, 151, 146.2 (d, *J*=5.67 Hz), 140.10, 134.6, 121.1, 106.7, 106.5 (d, *J*=7.99 Hz), 63.1, 52.0, 46.6, 41.3, 9.5. HRMS *m/z* calcd for $C_{18}H_{20}FN_3O_4$ $[M+H]^+$ 362.1516; found 362.1512.

9-fluoro-3-methyl-7-oxo-10-(piperazin-1-yl)-2,3-dihydro-7H-[1,4]oxazino[2,3,4-ij]quinoline-6-carboxylic acid (16):

Piperazine (0.184 g, 2.14 mmol) was added to a suspension of 9,10-difluoro-2,3-dihydro-3-methyl-7-oxo-7H-pyrido[1,2,3-de]-1,4-benzoxazine-6-carboxylic acid (0.1 g, 0.35 mmol) in DMSO (1.5 mL). The mixture was stirred at 95 °C for 12 hr. The solvent was removed under vacuum and cold acetone (10 mL) was added to the mixture. The residue was triturated in acetone (4 mL) to provide **16** (87.8 mg, 72%) as a brown solid. Mp = 198±2°C. IR (KBr, ν cm^{-1}): 3366 (OH/NH), 2978 and 2910 (CH_3), 1664 (C=O), 1510 (ArC=C), 1430 (ArC=C), 1200 (NR_3). ^1H NMR (DMSO-d_6) δ ppm: 8.97 (s, 1H), 7.59 (d, *J* = 12.3 Hz, 1H), 4.92 (d, *J* = 6.3 Hz, 1H), 4.59 (d, *J* = 11.4 Hz, 1H), 4.59 (d, *J* = 11.4 Hz, 1H), 3.27 – 3.22 (m, 4H), 2.84 (d, *J* = 7.7 Hz, 4H), 1.45 (d, *J* = 6.7 Hz, 3H). ^{13}C NMR (DMSO-d_6) δ ppm: 206.5, 173.8, 165.7, 164.6, 146.6, 141 (d, *J*=7.04 Hz), 123.8, 109.6, 107.6 (br s), 106.2, 103.1, 67.6, 54.8, 51.5, 48.6, 46.2, 44.9, 30.7, 17.9. HRMS *m/z* calcd for $C_{27}H_{26}FN_4O_4$ $[M+H]^+$ 348.1359; found 348.1567.

B. Iron chelating properties of compounds 5a-d

Compounds **5a-d** do not present a free hydroxyl function in the quinoline moiety. Thus, they probably only present the previously described fluoroquinolones chelating properties, i.e. through ciprofloxacin carboxylate and keto groups.[13] Compound **5a** (R=Me) was selected as a model in this family of compounds and its ability to complex iron(III) was evaluated to verify this idea. We used UV-visible spectrophotometry, as already performed for compound **1**.

Figure S1 shows absorbance spectra of compound **5a** in presence of iron(III) at pH 2. The addition of $FeCl_3$ to a solution of **5a** leads to a red shift of the π-π* band from 284 to 296 nm and the appearance of a LMCT band at 450 nm as observed for compound **1**, suggesting iron-chelation through its fluoroquinolone part only. Besides, the spectrum also shows one isosbestic point at 344 nm, indicating the formation of a single iron complex. In addition, when the absorbance at 450 nm was plotted against the ratio [Fe(III)]/[**5a**], we observed an increase in the absorbance, followed by a plateau. The two asymptotes intersect at a ratio of 1, implying a 1:1 stoichiometry (metal-ligand) for the complex. The affinity constant of the complex at pH 2 was determined using Specfit analysis of the spectra. The low value obtained (log K_{11} = 2.6 ± 0.2) is close to that of compound **1** (log K_{11} = 2.5 ± 0.3). These results show that iron-chelating ability at pH 2.0 of compounds **1** and **5a** are similar (Table S1), and as expected, confirm that the iron chelation occurs through by the fluoroquinolone part of **5a**.

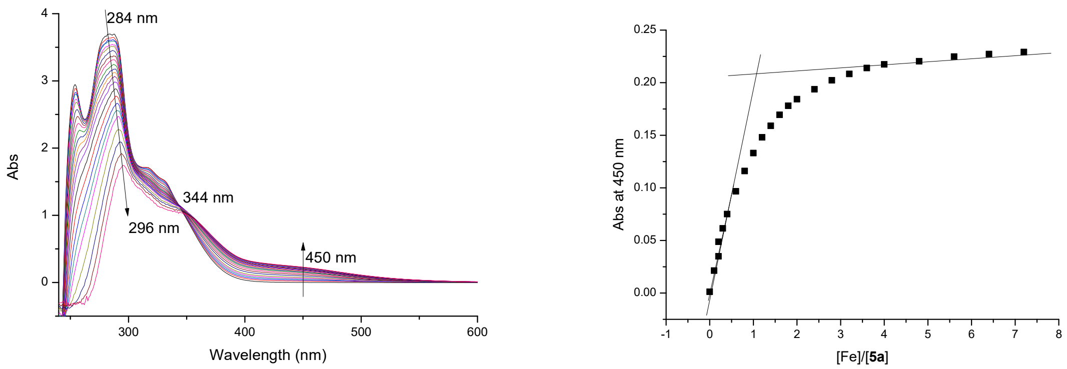

Figure S1. Absorption spectra in the presence of increasing concentrations of $FeCl_3$ (0 - 672 μM) at pH 2 and 25±0.5 °C

(A) of compound **5a** (10^{-4} M) (solvent: DMSO/water pH 2, 1:1, v/v); (B) Absorbance at 450 nm plotted against [Fe(III)]/[**5a**].

**Compound**	**π-π* (nm)**	**Isosbestic point (nm)**	**LMCT (nm)**	**log K_{11}**
**5a**	284 - 296	344	450	2.6 ± 0.2
**1**	284 - 290	340	450	2.5 ± 0.3

Table S1. Characterization of absorption spectra of compound **5a** and **1** at pH 2.0

C. Iron chelating properties of compounds 6 at pH 2.

At pH 2, the addition of $FeCl_3$ to a solution of **6** leads to a hypsochromic shift of the π-π* band from 284 to 280 nm (concentration of Fe^{3+}, from 0 to 196 μM) and then a bathochromic shift (red shift) of the π-π* band from 280 to 285 nm (from 196 μM to 385 μM), as well as the appearance of two ligand-to-metal charge-transfer (LMCT) bands at 450 nm and 600 nm (Figure S2). Furthermore, an isosbestic point is observed at 352 nm and is corresponding to the formation of a single iron complex. The plots of the absorbance at 450 and 600 nm against the [Fe^{3+}]/[**6**] ratio show increases in the absorbance, followed by a plateau (Figure S2). For each plot, the asymptotes intersect at a ratio of 1, implying the formation of iron complex with the stoichiometry of one metal for one ligand. The SPECFIT analysis of the spectra allows the determination of log K_{11} value of 2.8 ± 0.2.

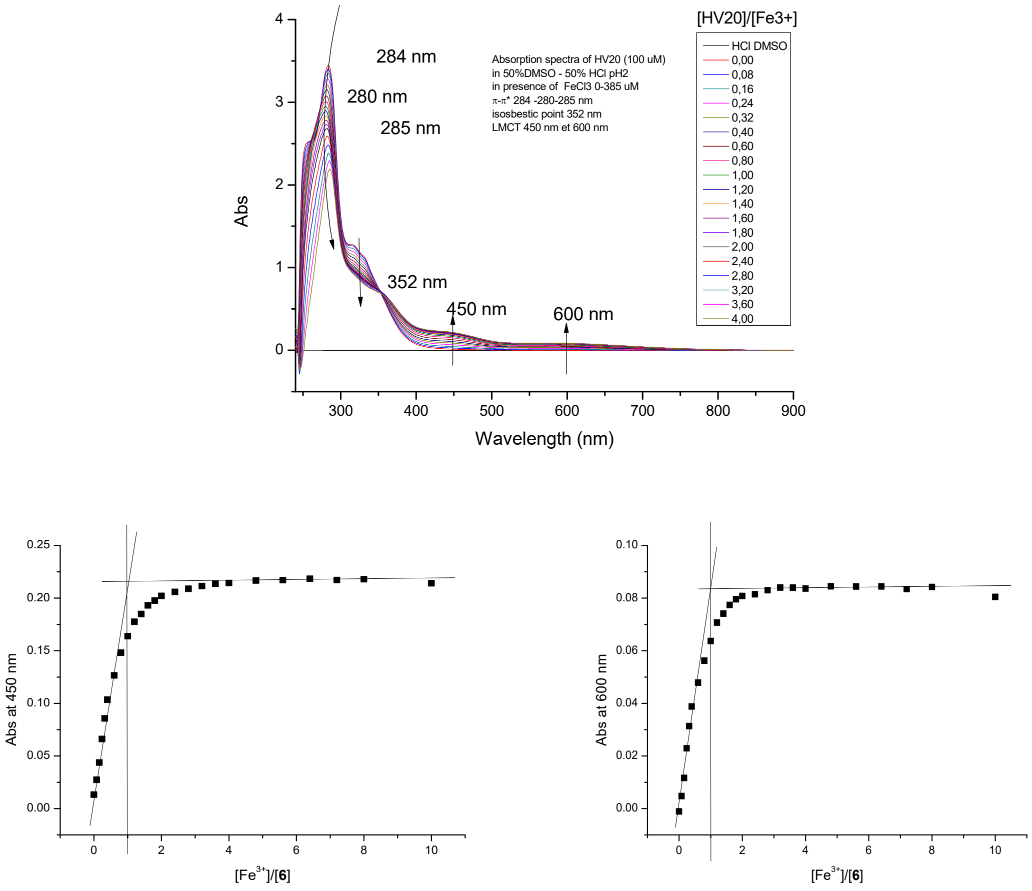

Figure S2. Absorption spectra in the presence of increasing concentrations of $FeCl_3$ (0-384 μM) of compound **6** (10^{-4} M) (solvent: DMSO/ water pH 2, 1:1 v/v; at pH 2 and 25±0.5 °C); Absorbance at 450 nm and 600 nm plotted against [Fe(III)]/[**6**].

D. Antichlamydial activity of the antimicrobials.

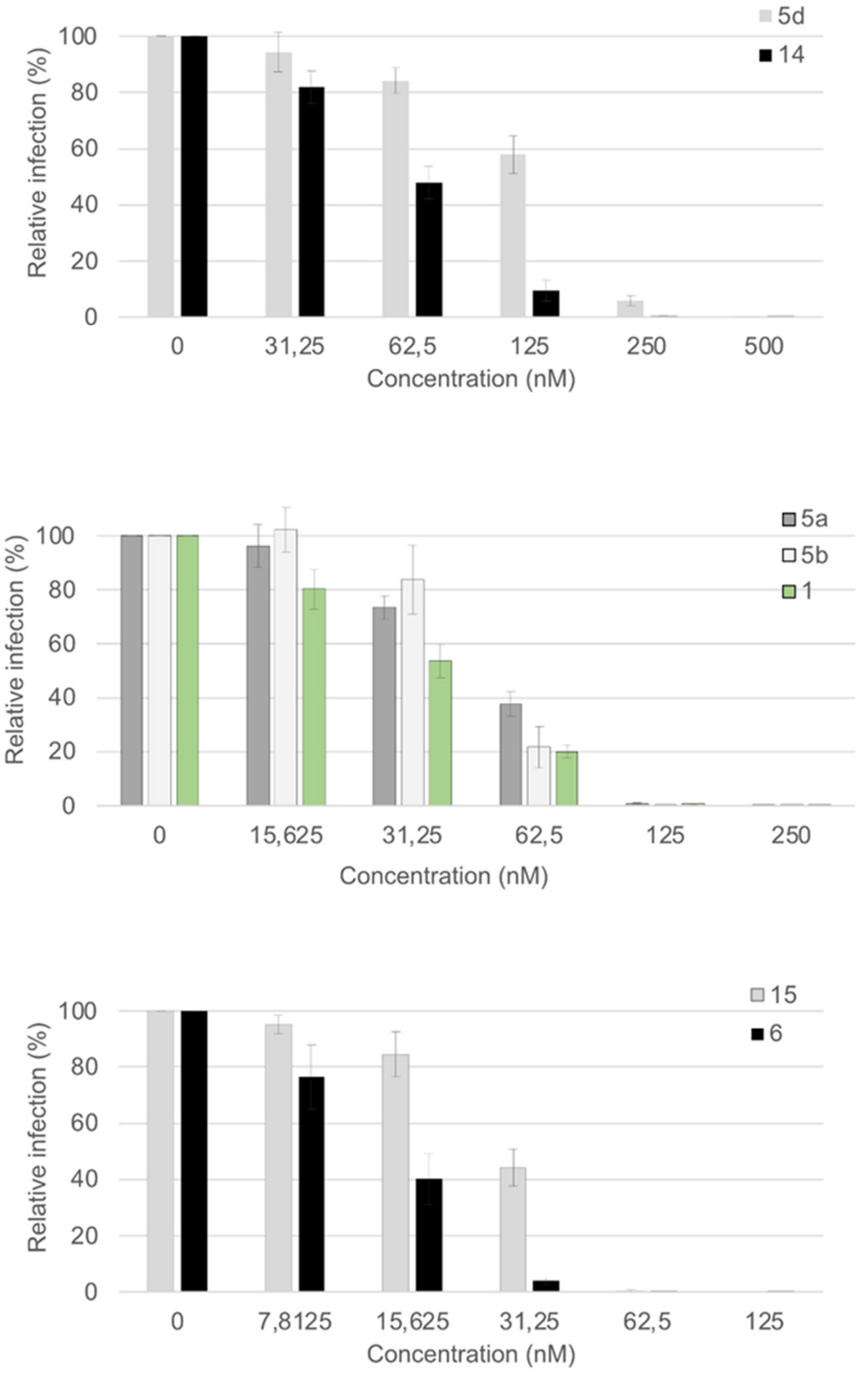

Figure S3. Dose response of the most potent compounds on *C. trachomatis* progeny reinfection for determination of MBC and EC_{50}. The number of *C. trachomatis* progeny from compound treated infections were normalized by setting DMSO treated infections to 100% for each experiment and calculating the relative infection for each compound concentration. Bars show means from at least three independent experiments and error bars depict standard deviation.

E. ¹H and ¹³C NMR of the final compounds

*C-F ($^{1}J_{CF}$, $^{2}J_{CF}$) coupling is illustrated for compound **1** using appropriate zoomed windows at the end of this document.*

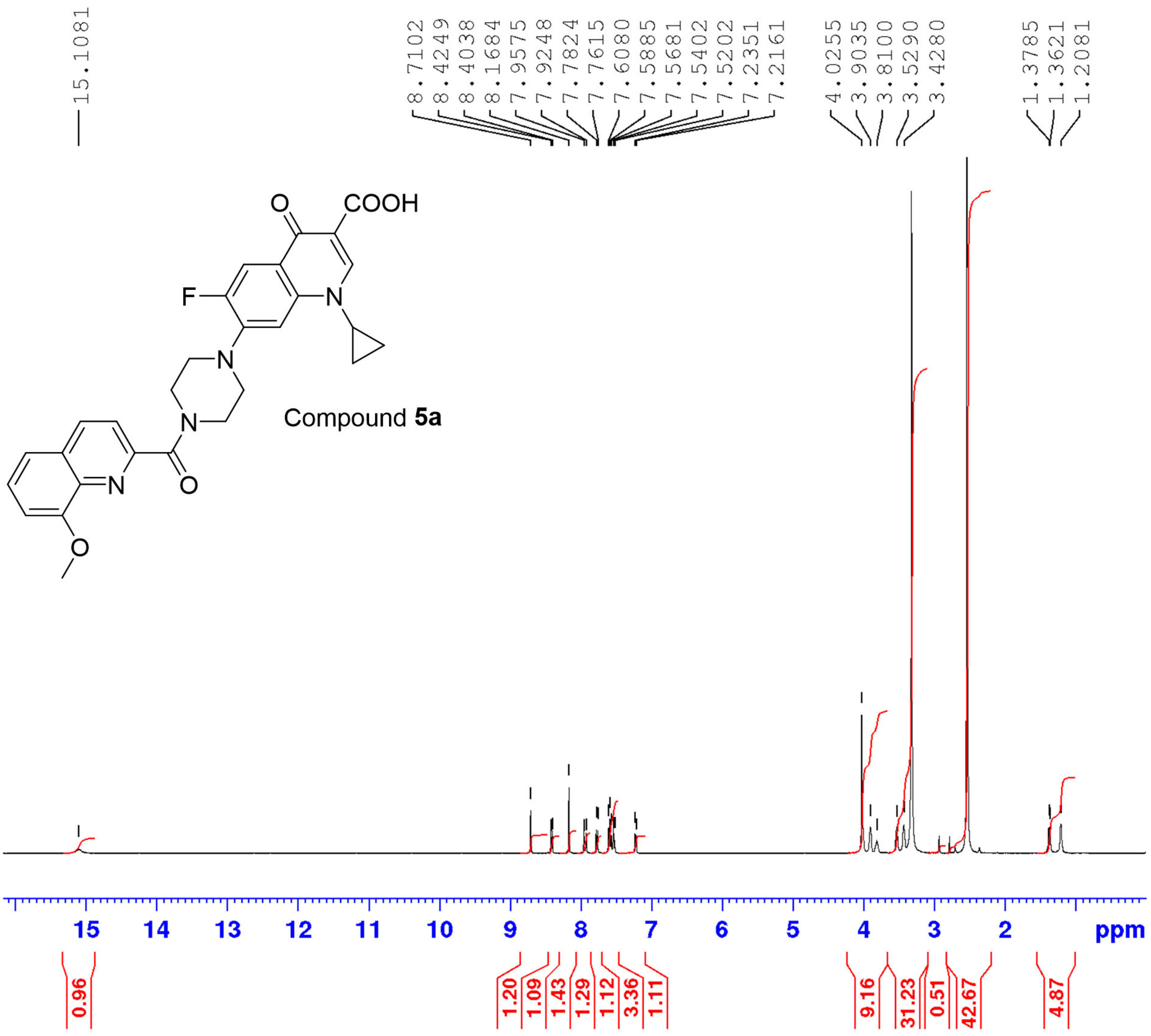

In DMSO-d₆

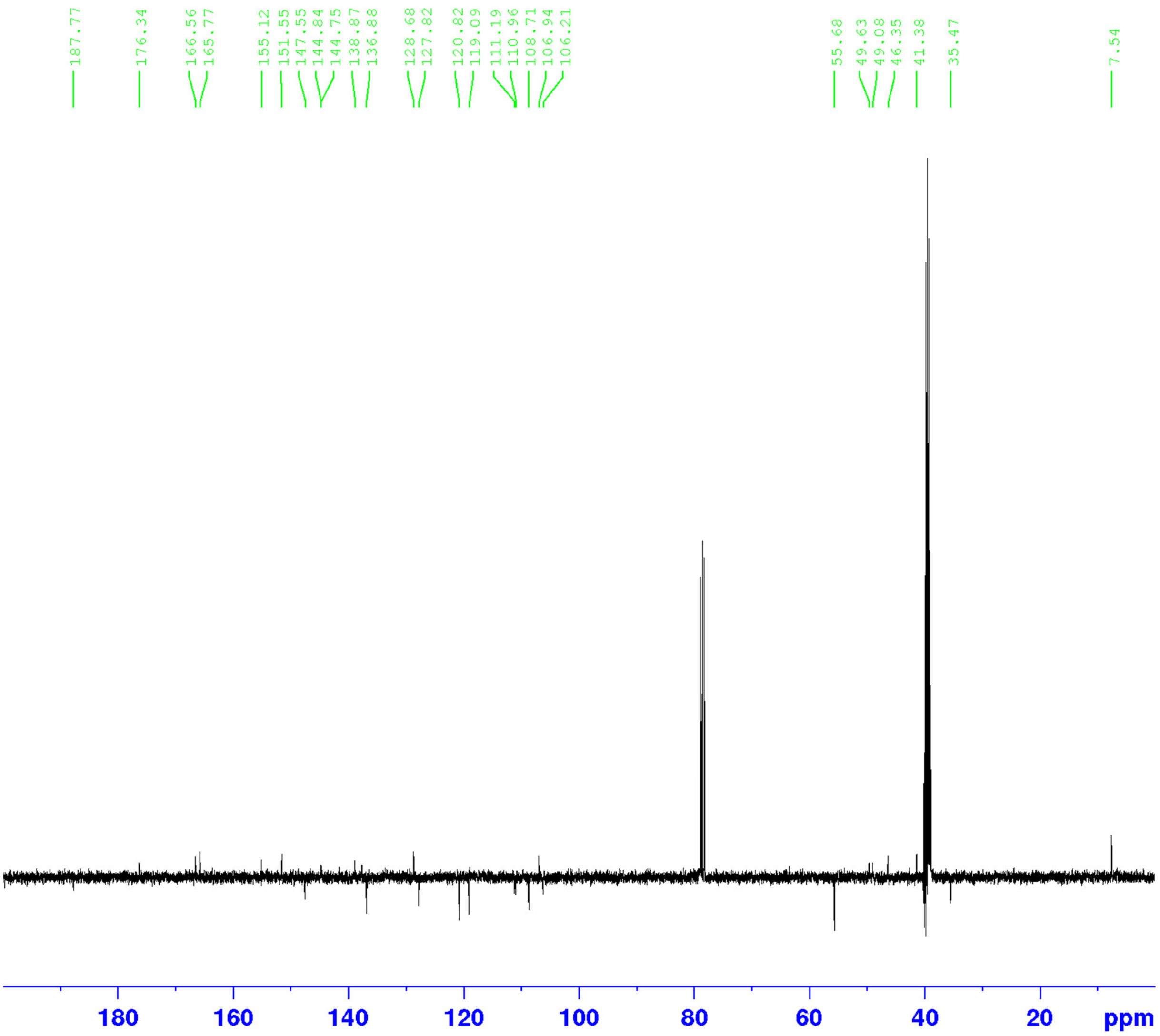
187.77
176.34
166.56
165.77
155.12
151.55
147.55
144.84
144.75
138.87
136.88
128.68
127.82
120.82
119.09
111.19
110.96
108.71
106.94
106.21
55.68
49.63
49.08
46.35
41.38
35.47
7.54
180
160
140
120
100
80
60
40
20
ppm

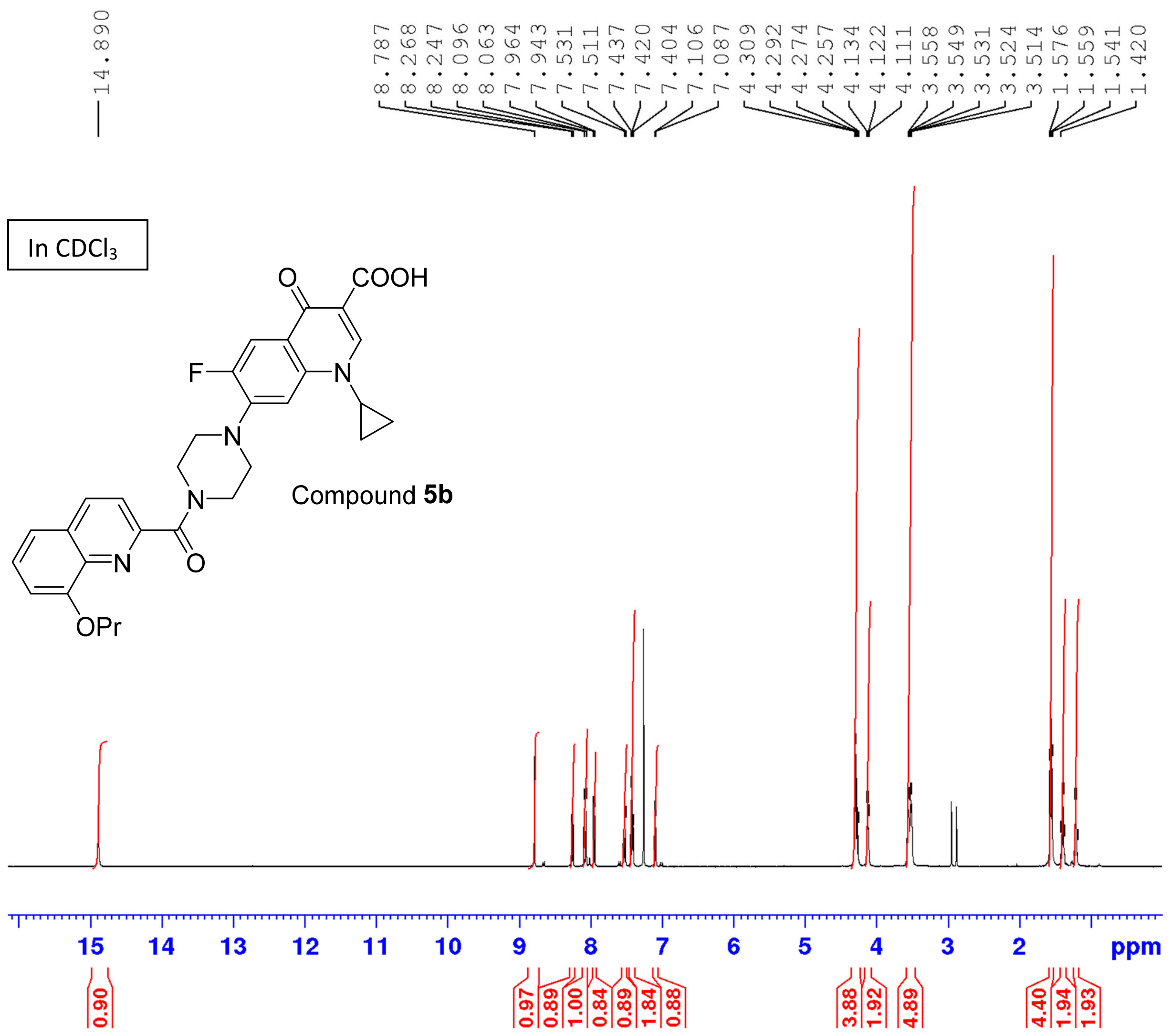
In CDCl$_3$
Compound **5b**
ppm

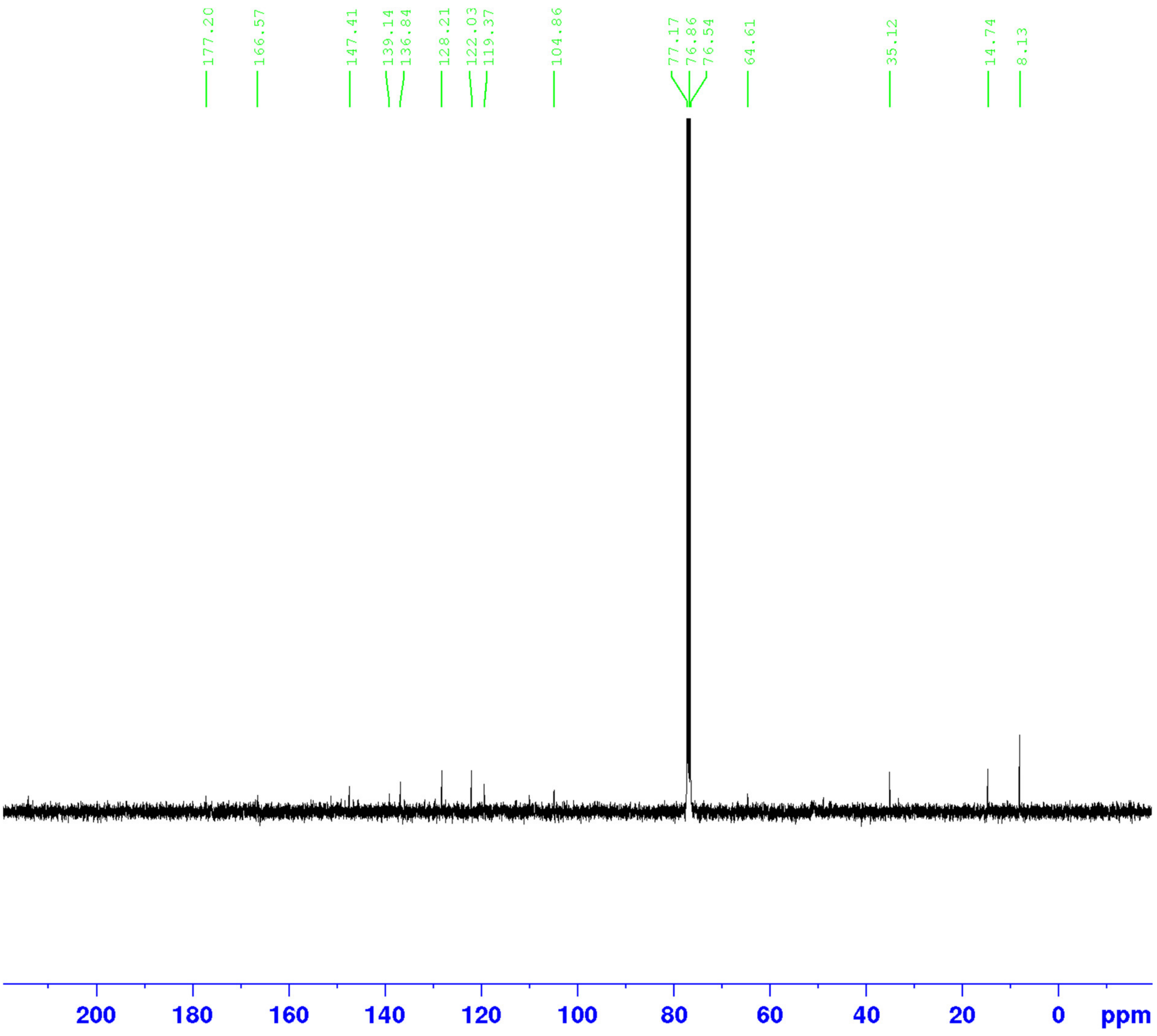
177.20
166.57
147.41
139.14
136.84
128.21
122.03
119.37
104.86
77.17
76.86
76.54
64.61
35.12
14.74
8.13
200
180
160
140
120
100
80
60
40
20
0
ppm

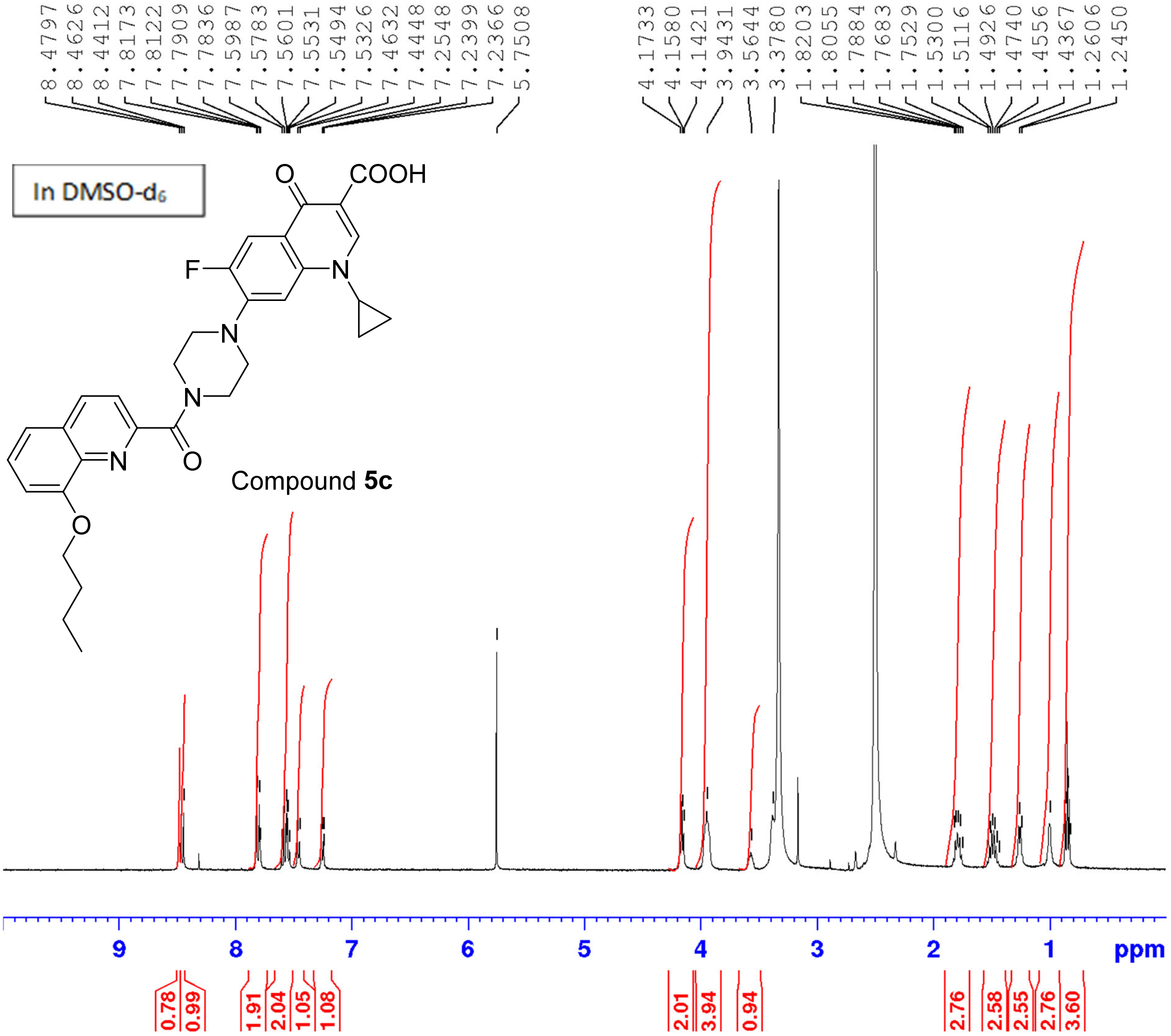
8.4797
8.4626
8.4412
7.8173
7.8122
7.7909
7.7836
7.5987
7.5783
7.5601
7.5531
7.5494
7.5326
7.4632
7.4448
7.2548
7.2399
7.2366
5.7508
4.1733
4.1580
4.1421
3.9431
3.5644
3.3780
1.8203
1.8055
1.7884
1.7683
1.7529
1.5300
1.5116
1.4926
1.4740
1.4556
1.4367
1.2606
1.2450
In DMSO-d6
COOH
O
F
N
N
N
N
O
O
Compound 5c
9
8
7
6
5
4
3
2
1
ppm
0.78
0.99
1.91
2.04
1.05
1.08
2.01
3.94
0.94
2.76
2.58
2.55
2.76
3.60

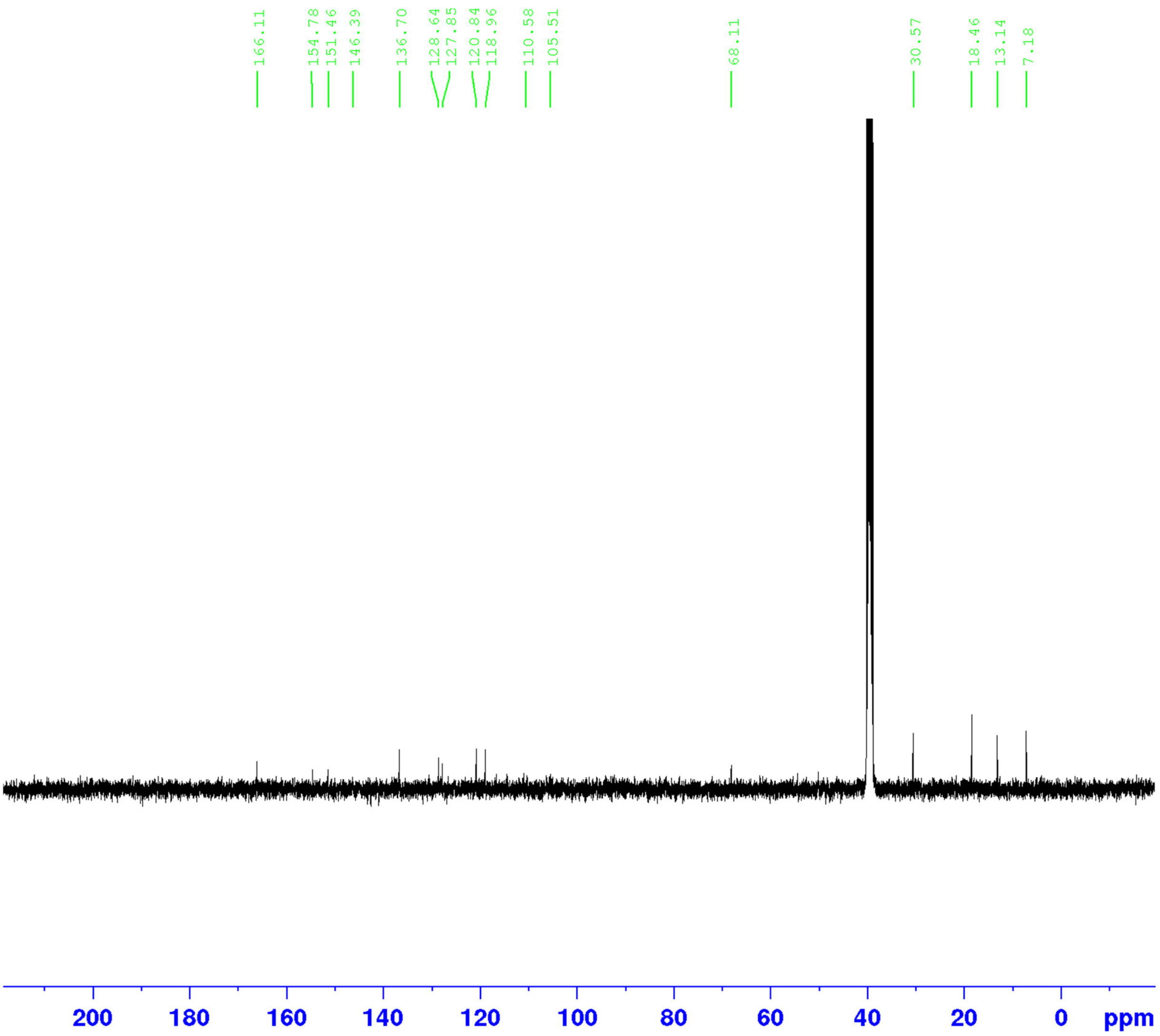
166.11
154.78
151.46
146.39
136.70
128.64
127.85
120.84
118.96
110.58
105.51
68.11
30.57
18.46
13.14
7.18
200
180
160
140
120
100
80
60
40
20
0
ppm

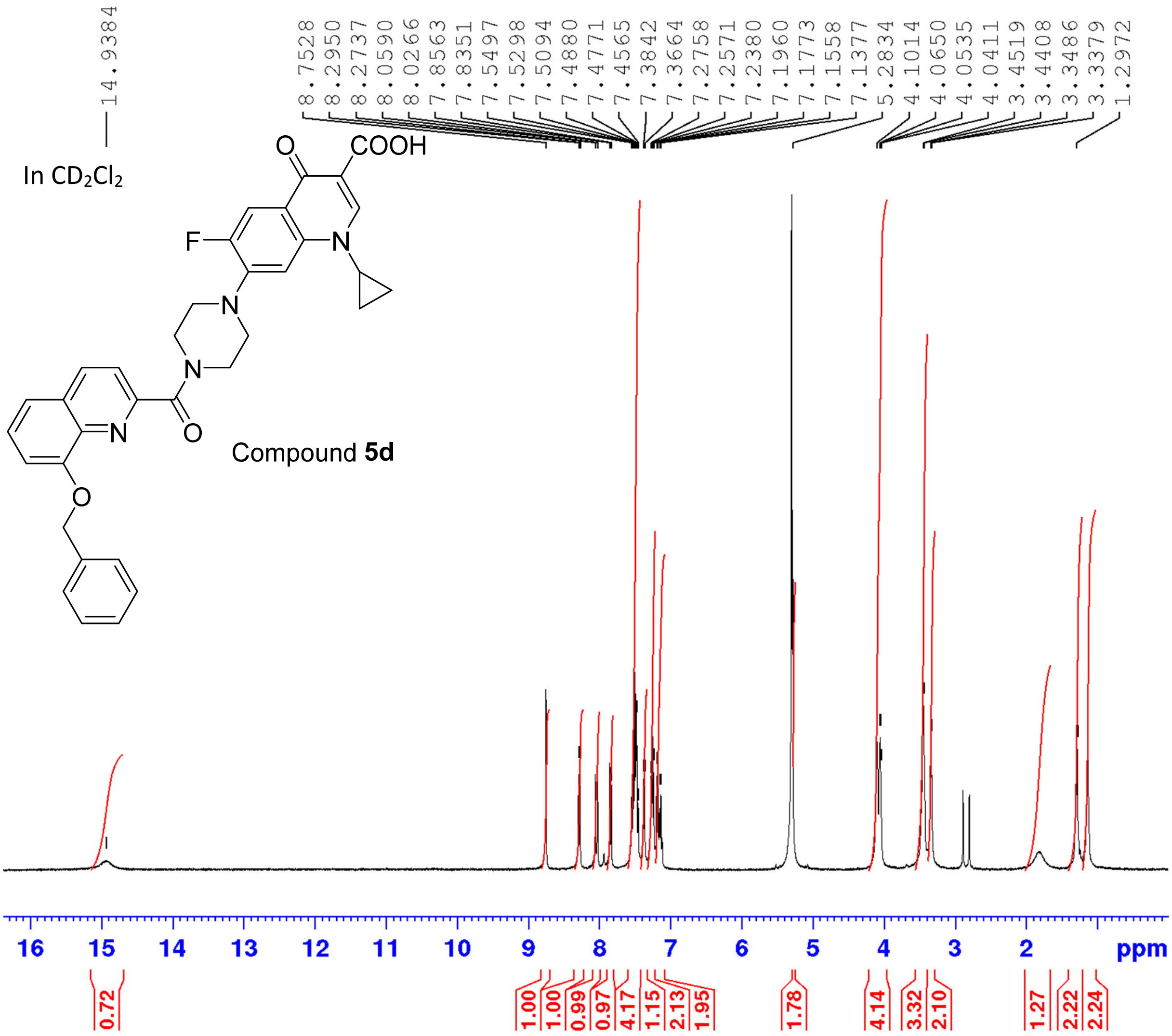

In CD2Cl2
14.9384
8.7528
8.2950
8.2737
8.0590
8.0266
7.8563
7.8351
7.5497
7.5298
7.5094
7.4880
7.4771
7.4565
7.3842
7.3664
7.2758
7.2571
7.2380
7.1960
7.1773
7.1558
7.1377
5.2834
4.1014
4.0650
4.0535
4.0411
3.4519
3.4408
3.3486
3.3379
1.2972
Compound 5d
16
15
14
13
12
11
10
9
8
7
6
5
4
3
2
ppm
0.72
1.00
1.00
0.99
0.97
4.17
1.15
2.13
1.95
1.78
4.14
3.32
2.10
1.27
2.22
2.24

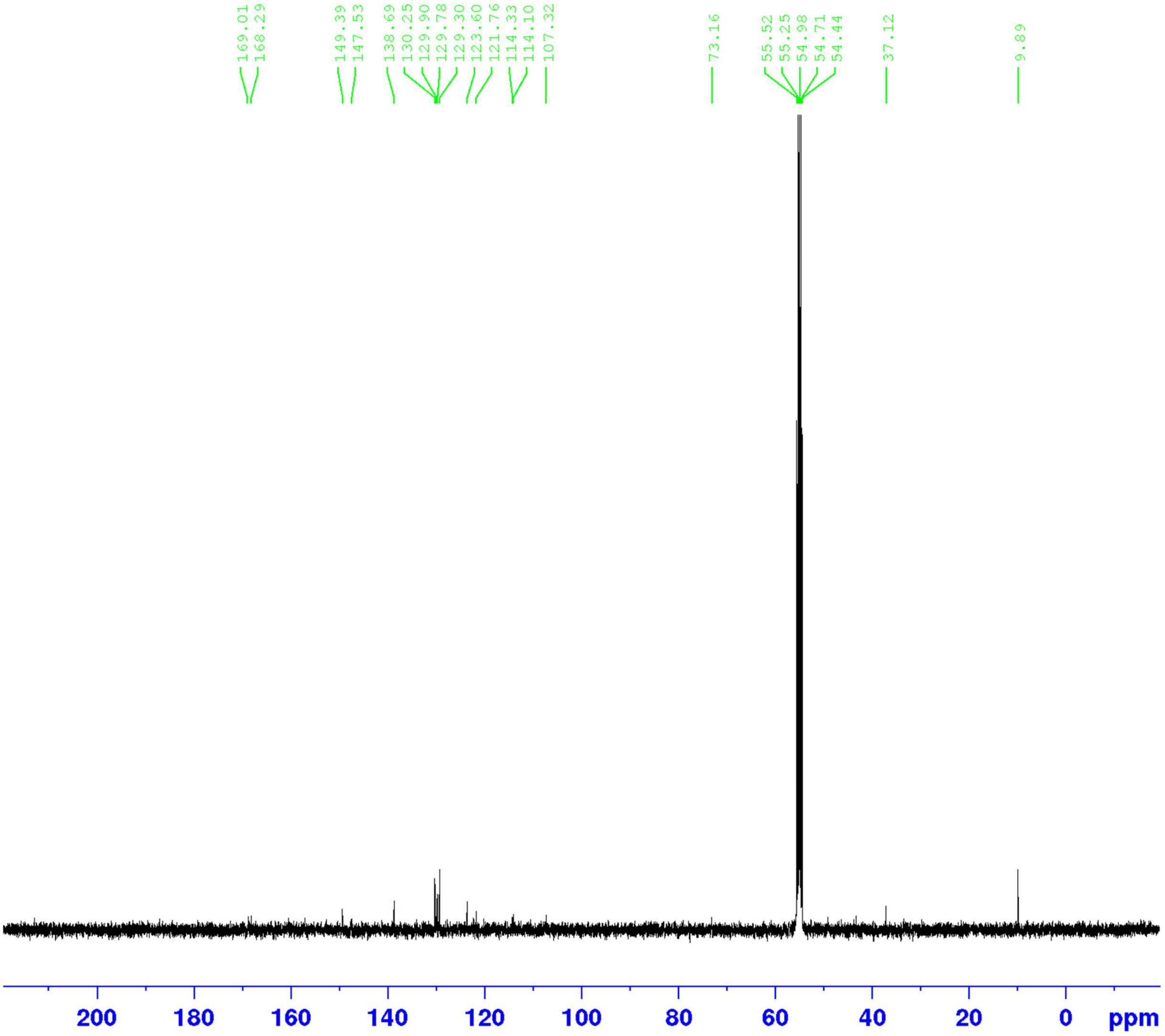
169.01
168.29
149.39
147.53
138.69
130.25
129.90
129.78
129.30
123.60
121.76
114.33
114.10
107.32
73.16
55.52
55.25
54.98
54.71
54.44
37.12
9.89
200
180
160
140
120
100
80
60
40
20
0
ppm

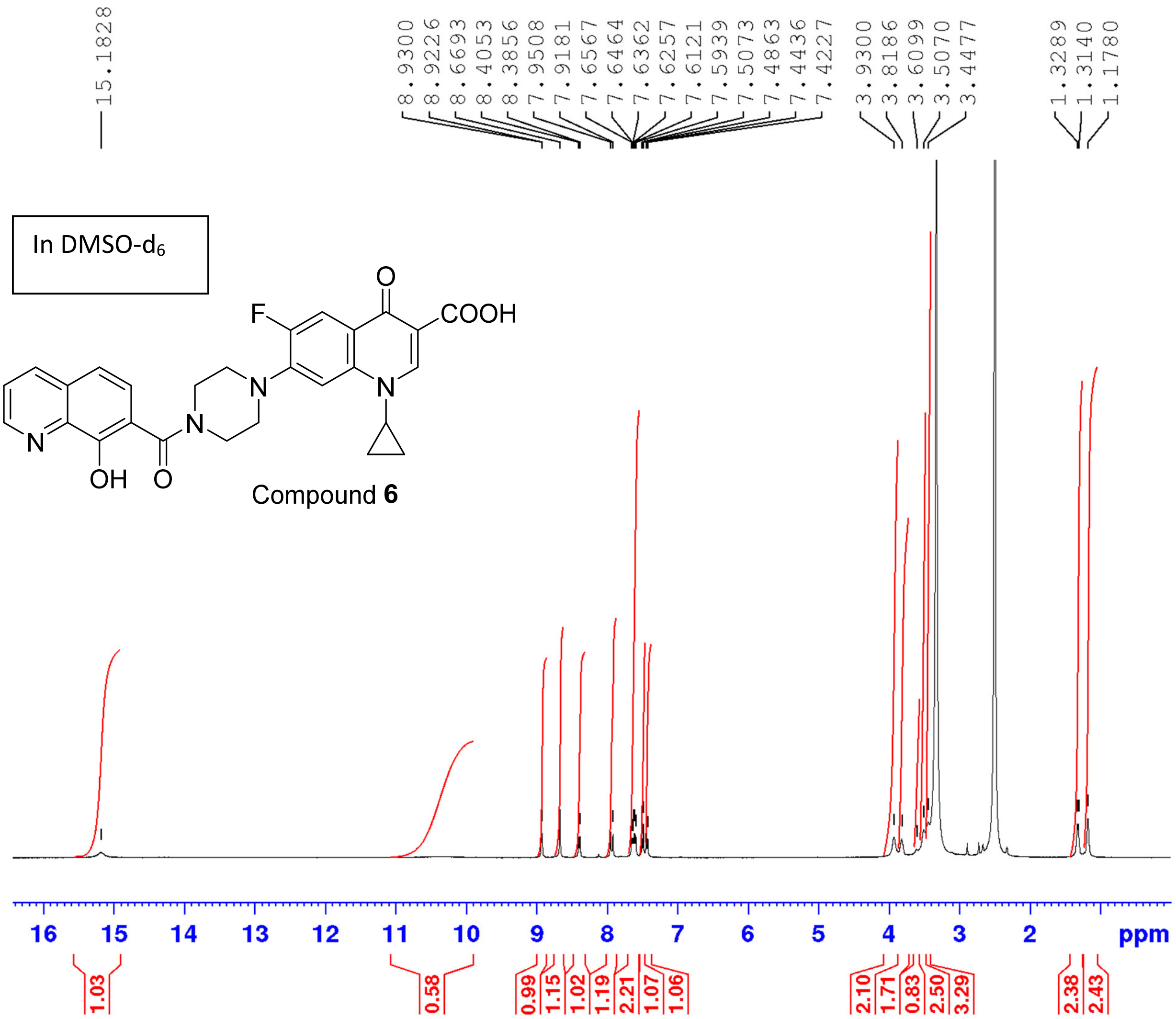
In DMSO-d6
F
O
COOH
N
N
N
N
OH
O
Compound 6
15.1828
8.9300 8.9226 8.6693 8.4053 8.3856 7.9508 7.9181 7.6567 7.6464 7.6362 7.6257 7.6121 7.5939 7.5073 7.4863 7.4436 7.4227
3.9300 3.8186 3.6099 3.5070 3.4477
1.3289 1.3140 1.1780
16 15 14 13 12 11 10 9 8 7 6 5 4 3 2 ppm
1.03 0.58 0.99 1.15 1.02 1.19 2.21 1.07 1.06 2.10 1.71 0.83 2.50 3.29 2.38 2.43

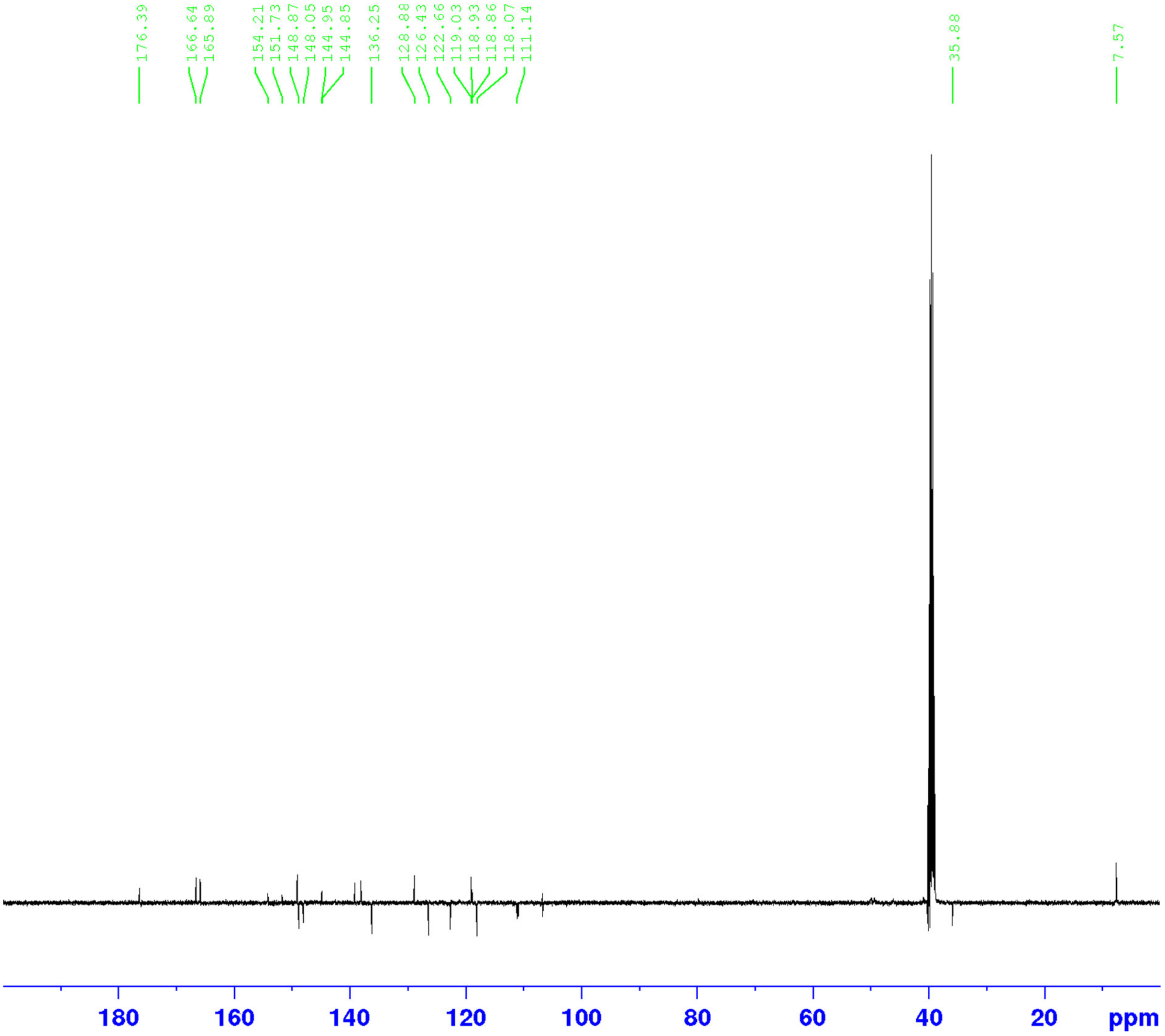

176.39
166.64
165.89
154.21
151.73
148.87
148.05
144.95
144.85
136.25
128.88
126.43
122.66
119.03
118.93
118.86
118.07
111.14
35.88
7.57
180
160
140
120
100
80
60
40
20
ppm

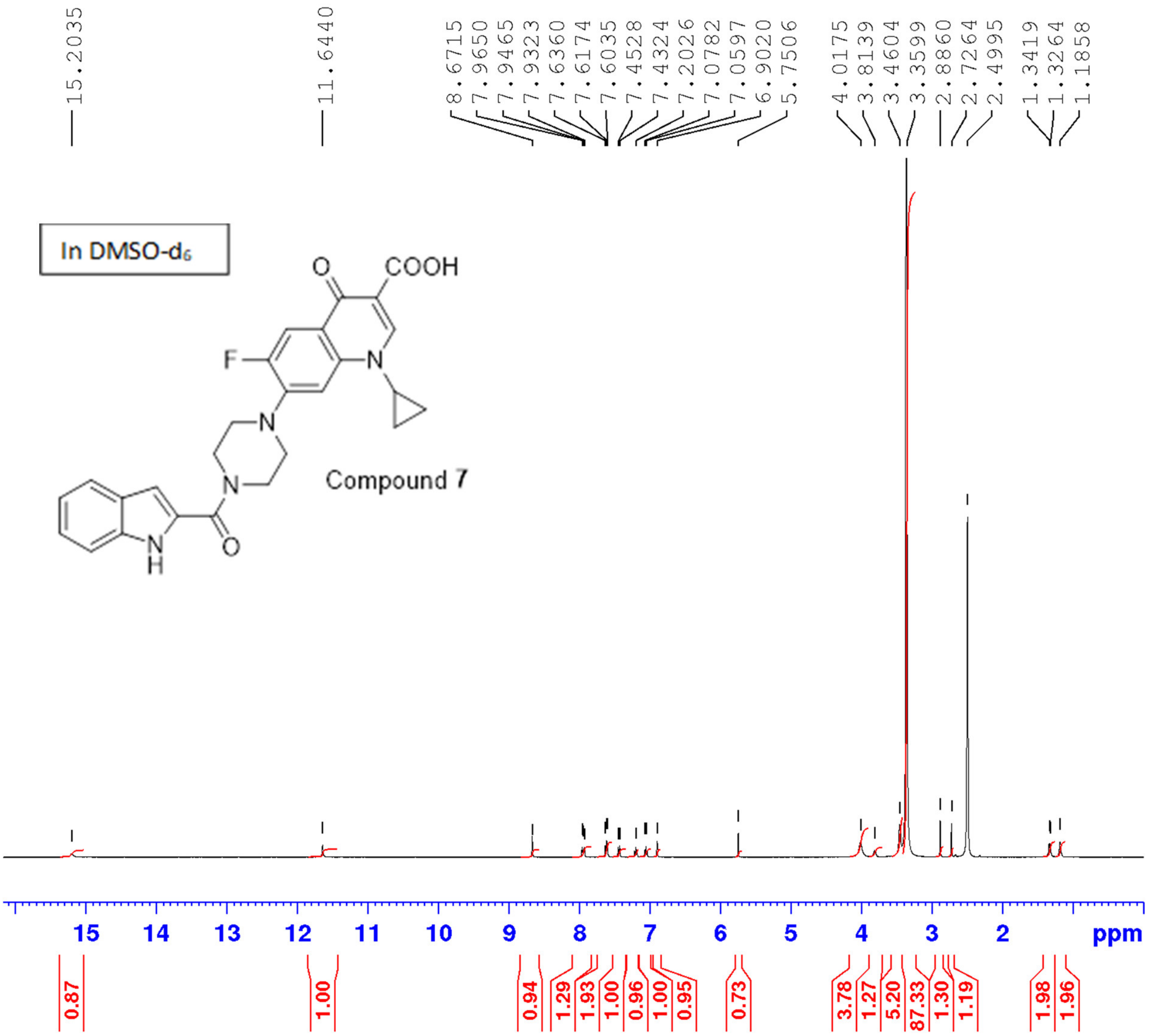
In DMSO-d6
Compound 7
COOH
15.2035
11.6440
8.6715
7.9650
7.9465
7.9323
7.6360
7.6174
7.6035
7.4528
7.4324
7.2026
7.0782
7.0597
6.9020
5.7506
4.0175
3.8139
3.4604
3.3599
2.8860
2.7264
2.4995
1.3419
1.3264
1.1858
15 14 13 12 11 10 9 8 7 6 5 4 3 2 ppm
0.87
1.00
0.94
1.29
1.93
1.00
0.96
1.00
0.95
0.73
3.78
1.27
5.20
87.33
1.30
1.19
1.98
1.96

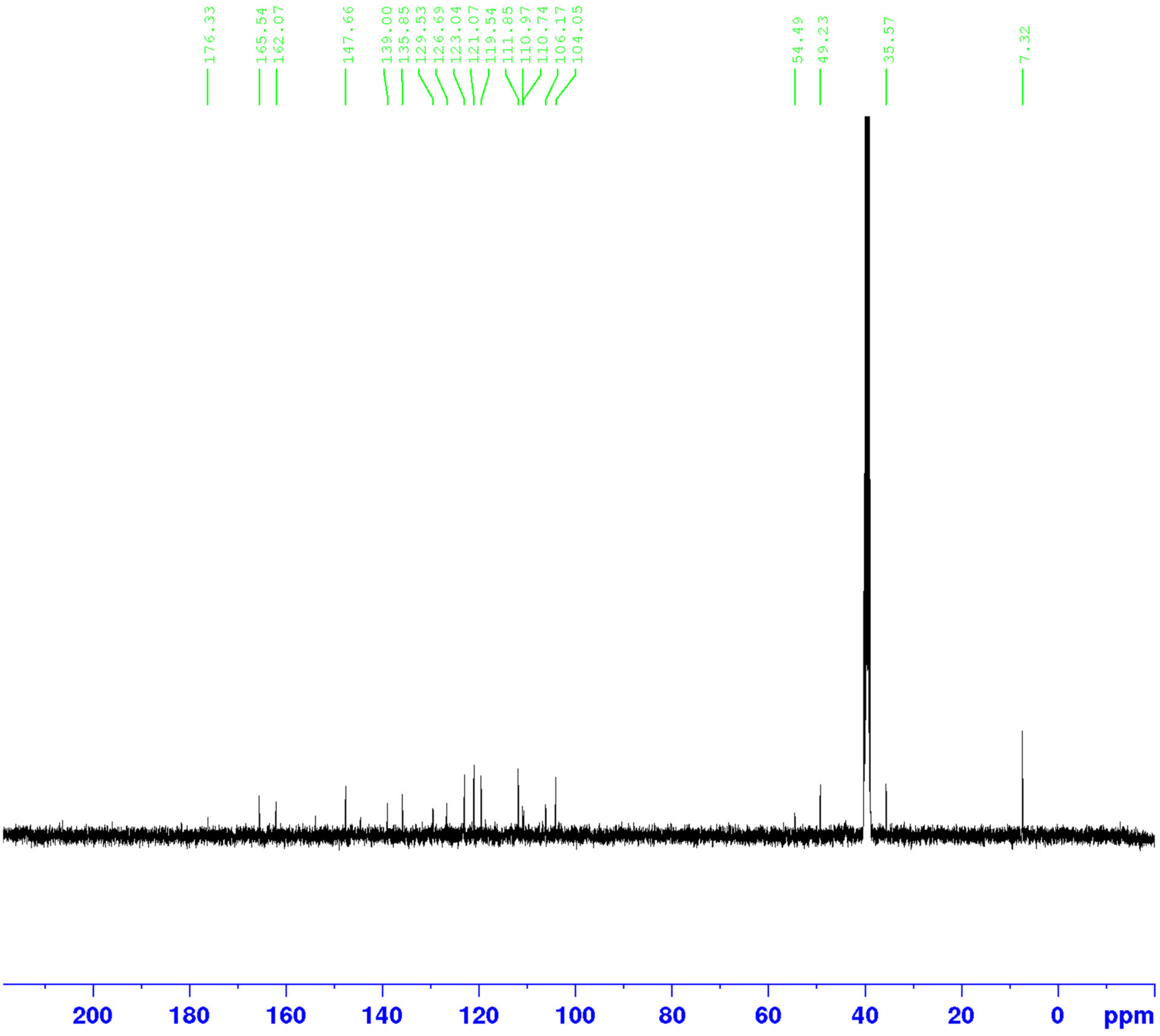
176.33
165.54
162.07
147.66
139.00
135.85
129.53
126.69
123.04
121.07
119.54
111.85
110.97
110.74
106.17
104.05
54.49
49.23
35.57
7.32
200
180
160
140
120
100
80
60
40
20
0
ppm

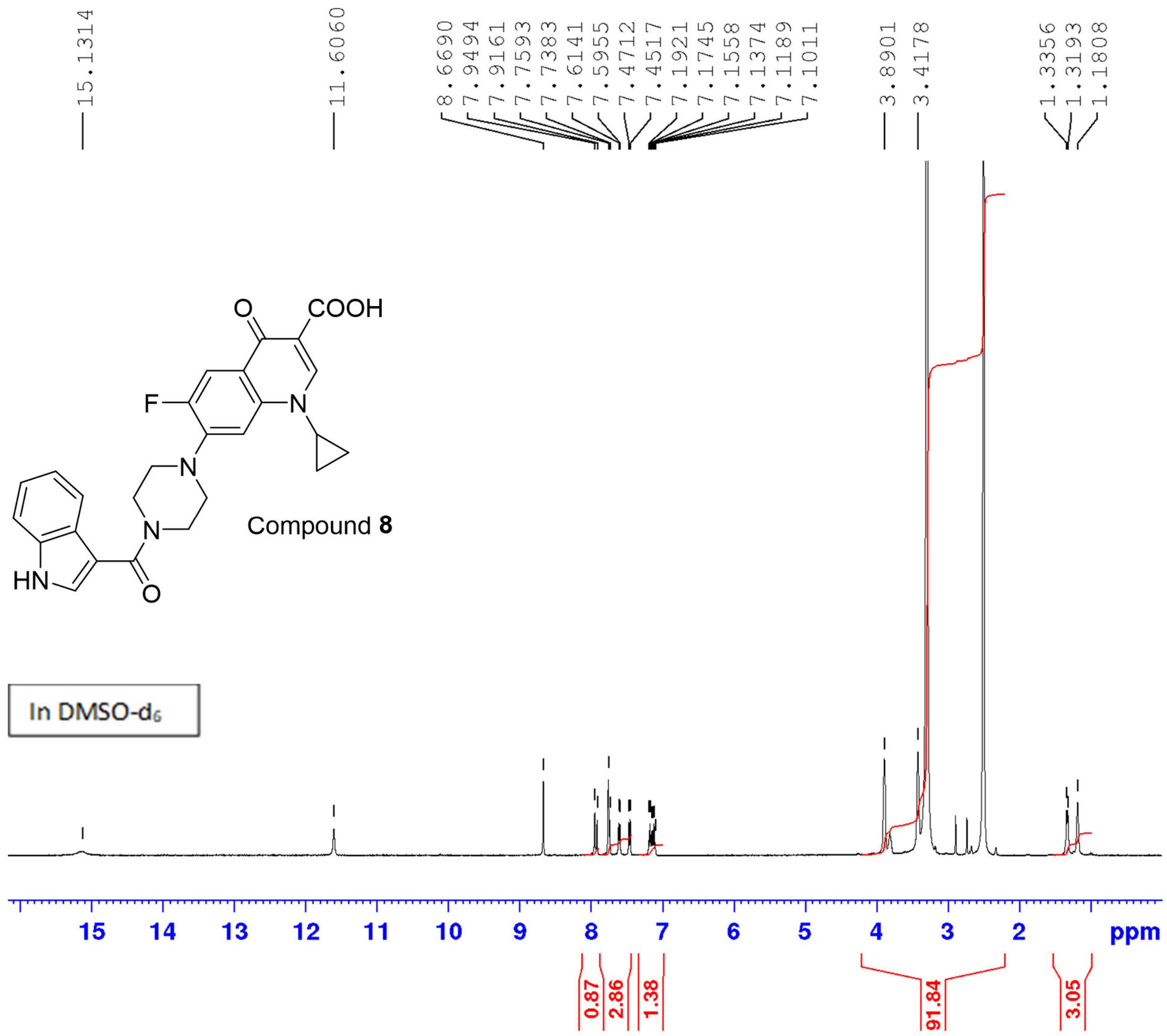
15.1314
11.6060
8.6690
7.9494
7.9161
7.7593
7.7383
7.6141
7.5955
7.4712
7.4517
7.1921
7.1745
7.1558
7.1374
7.1189
7.1011
3.8901
3.4178
1.3356
1.3193
1.1808
COOH
O
F
N
N
N
HN
O
Compound 8
In DMSO-d6
15
14
13
12
11
10
9
8
7
6
5
4
3
2
ppm
0.87
2.86
1.38
91.84
3.05

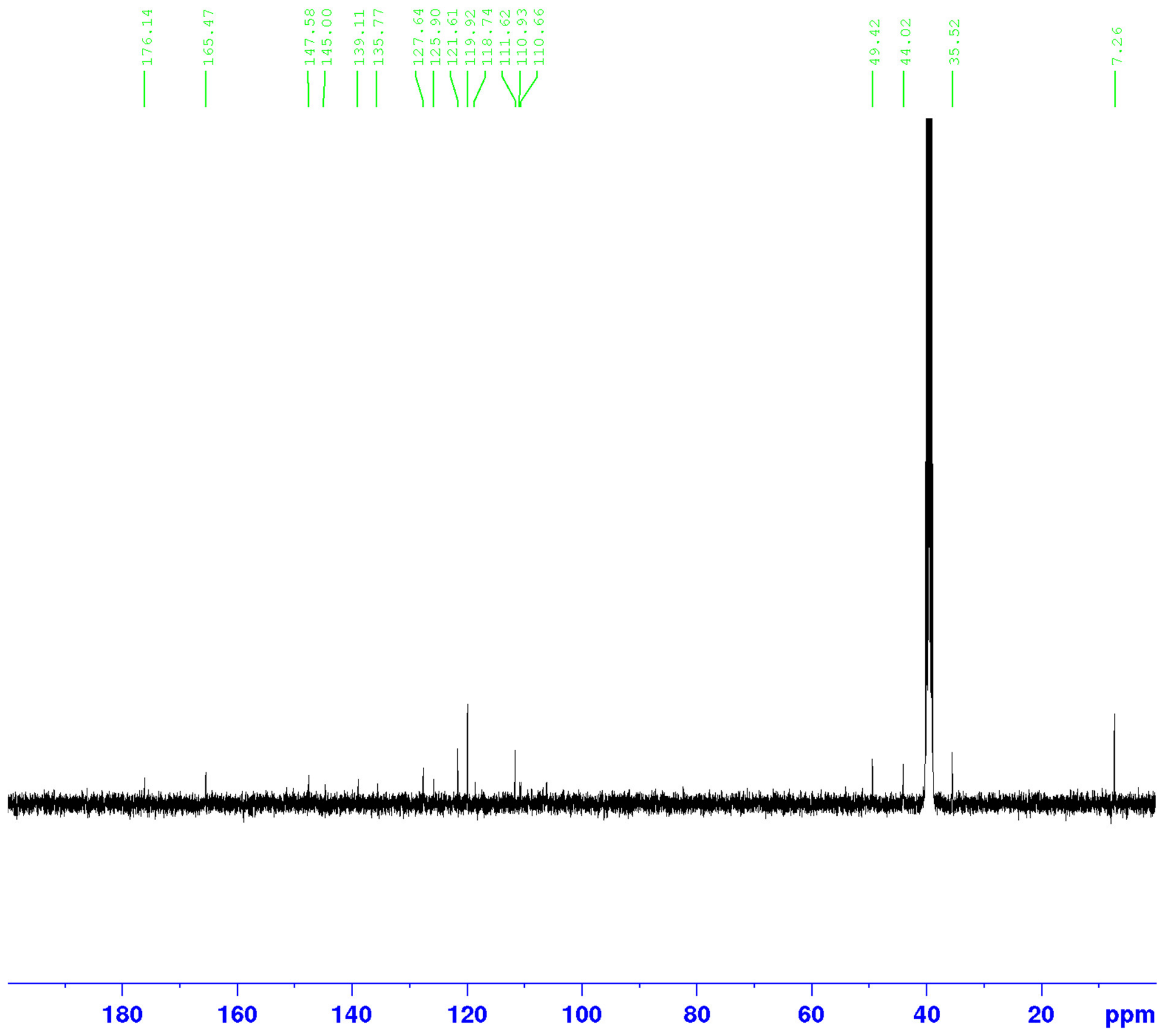
176.14
165.47
147.58
145.00
139.11
135.77
127.64
125.90
121.61
119.92
118.74
111.62
110.93
110.66
49.42
44.02
35.52
7.26
180
160
140
120
100
80
60
40
20
ppm

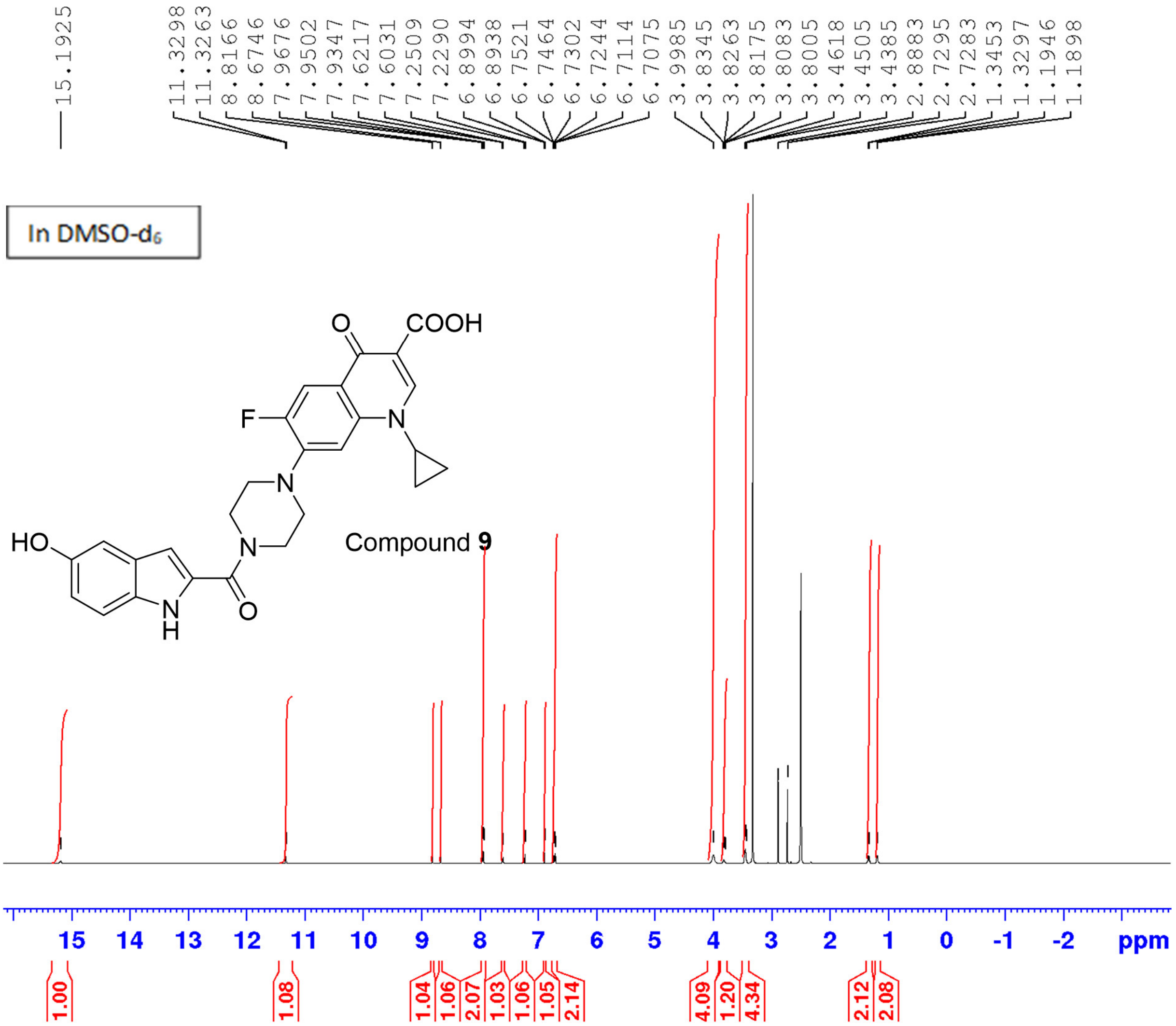
In DMSO-d6
Compound 9
ppm

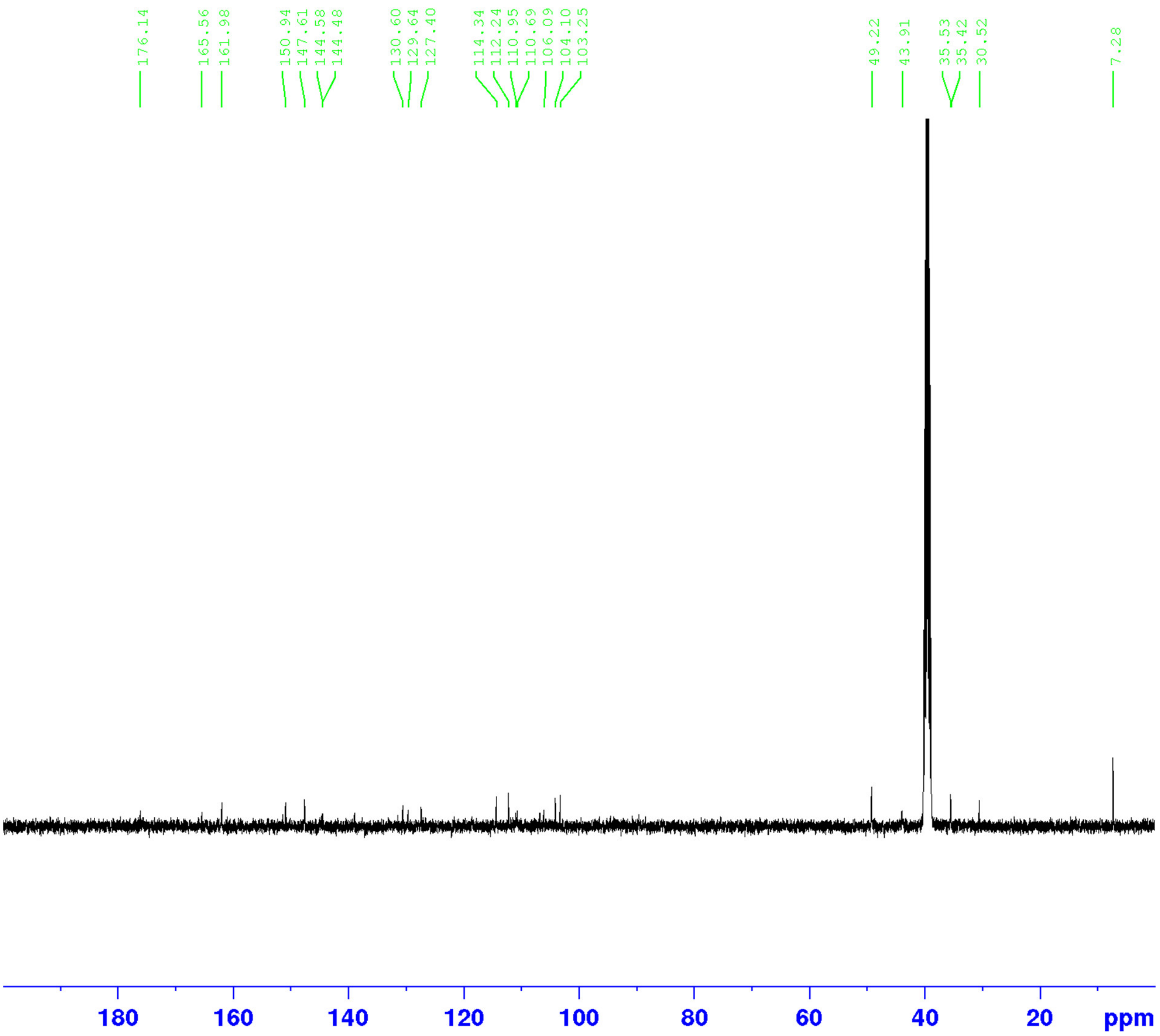
176.14
165.56
161.98
150.94
147.61
144.58
144.48
130.60
129.64
127.40
114.34
112.24
110.95
110.69
106.09
104.10
103.25
49.22
43.91
35.53
35.42
30.52
7.28
180
160
140
120
100
80
60
40
20
ppm

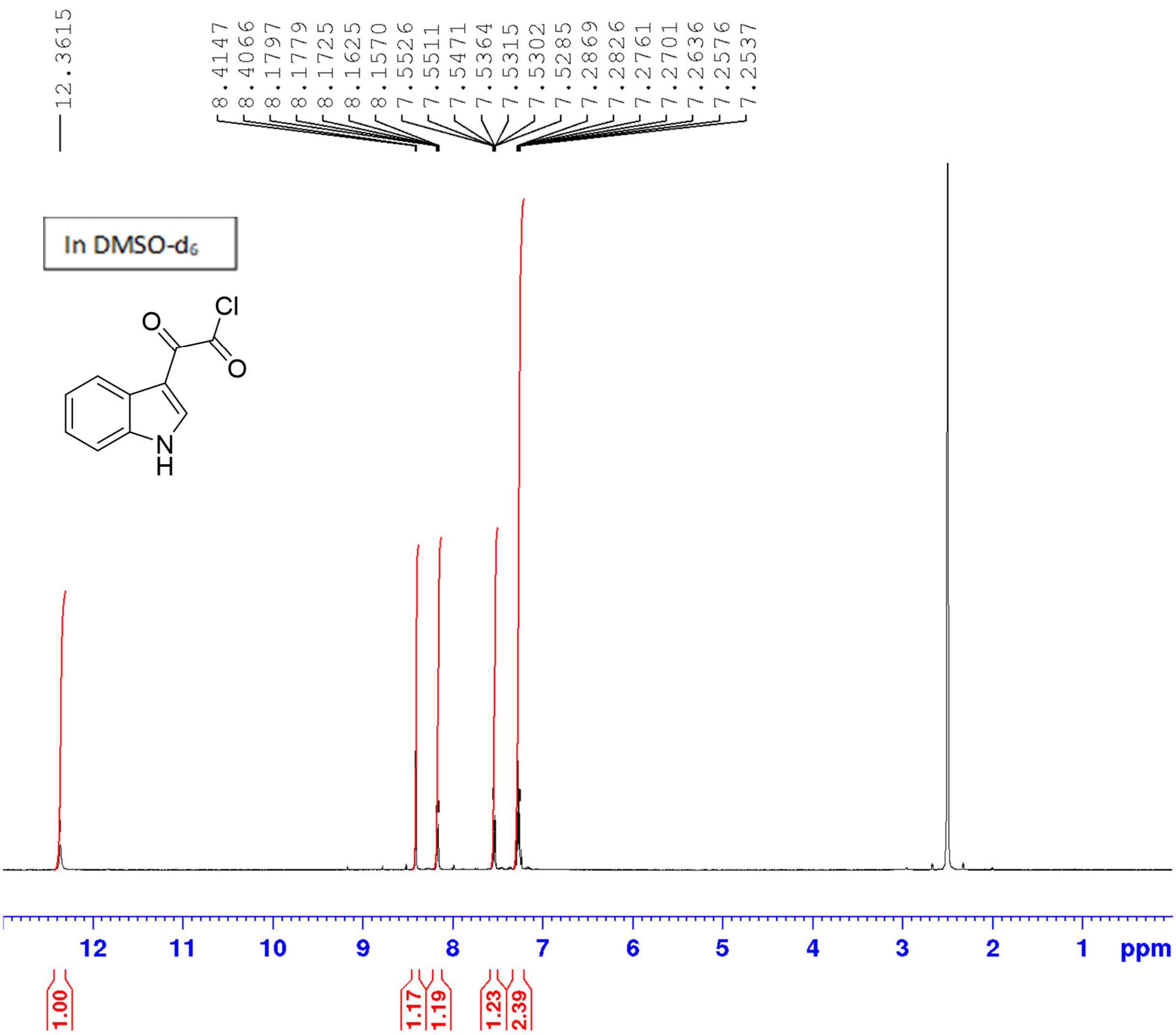
12.3615
8.4147
8.4066
8.1797
8.1779
8.1725
8.1625
8.1570
7.5526
7.5511
7.5471
7.5364
7.5315
7.5302
7.5285
7.2869
7.2826
7.2761
7.2701
7.2636
7.2576
7.2537
In DMSO-d6
Cl
O
O
N
H
12
11
10
9
8
7
6
5
4
3
2
1
ppm
1.00
1.17
1.19
1.23
2.39

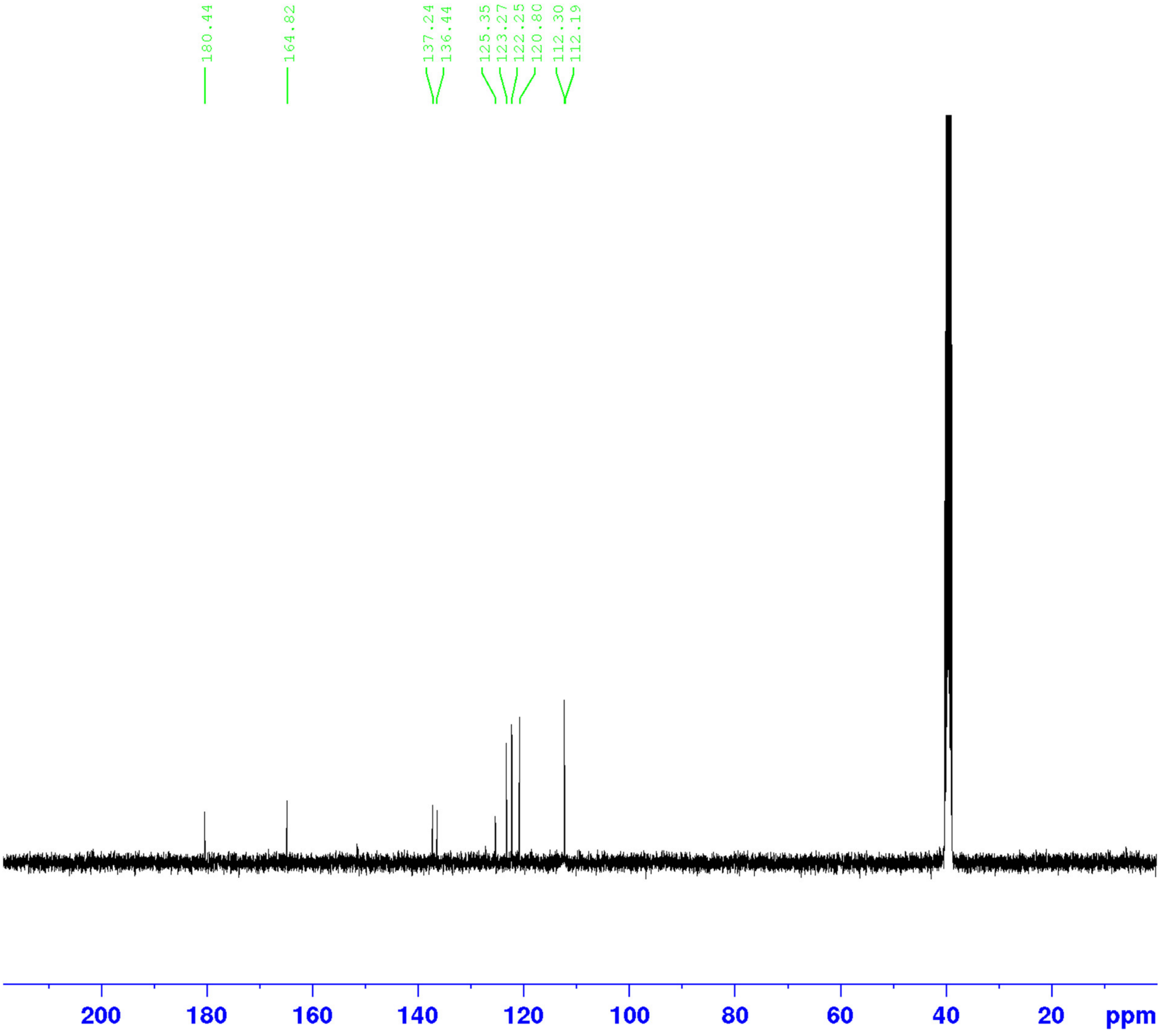
180.44
164.82
137.24
136.44
125.35
123.27
122.25
120.80
112.30
112.19
200
180
160
140
120
100
80
60
40
20
ppm

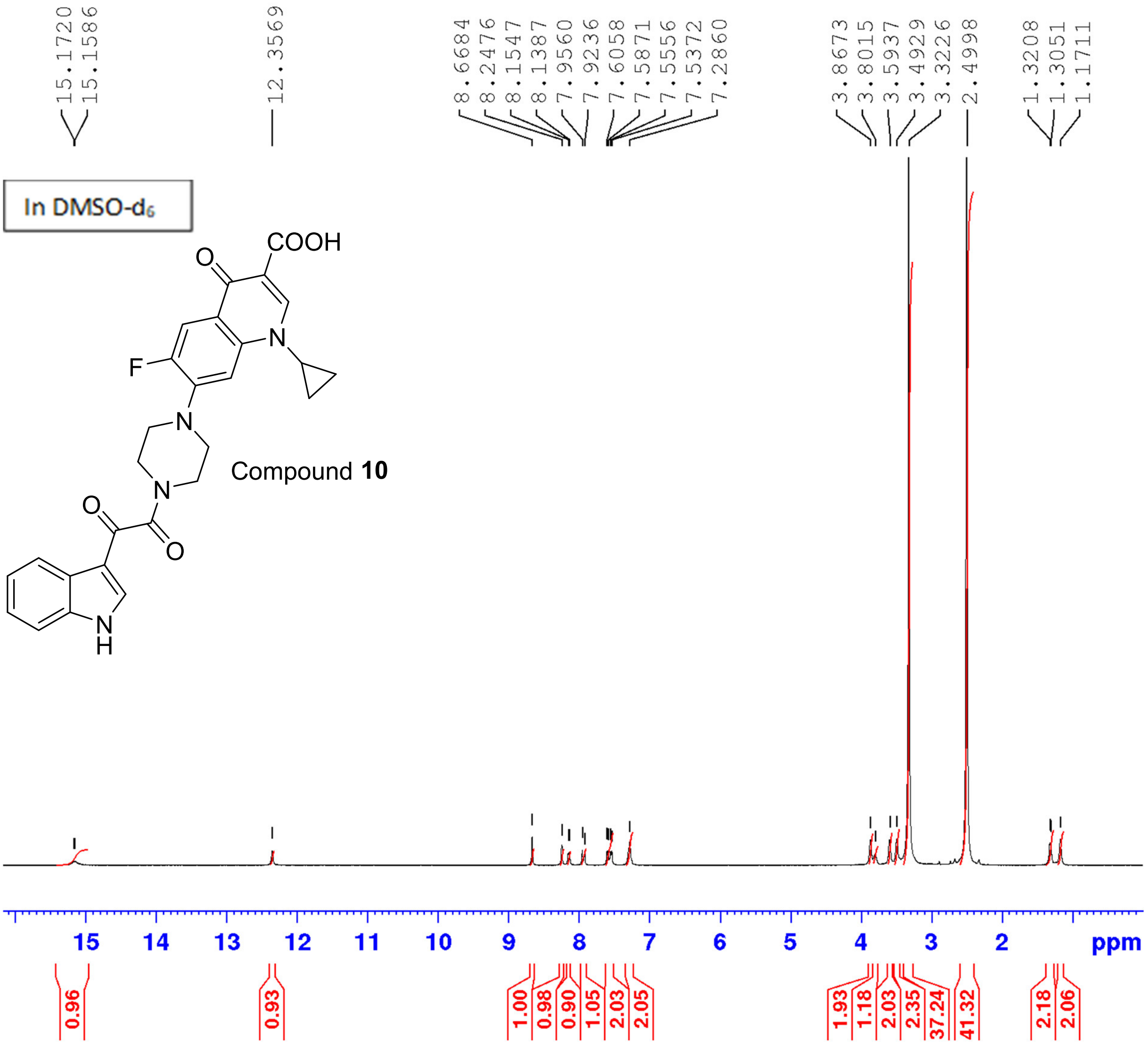

15.1720
15.1586
12.3569
8.6684
8.2476
8.1547
8.1387
7.9560
7.9236
7.6058
7.5871
7.5556
7.5372
7.2860
3.8673
3.8015
3.5937
3.4929
3.3226
2.4998
1.3208
1.3051
1.1711
In DMSO-d6
COOH
O
F
N
N
N
O
O
N
H
Compound 10
15 14 13 12 11 10 9 8 7 6 5 4 3 2 ppm
0.96
0.93
1.00
0.98
0.90
1.05
2.03
2.05
1.93
1.18
2.03
2.35
37.24
41.32
2.18
2.06

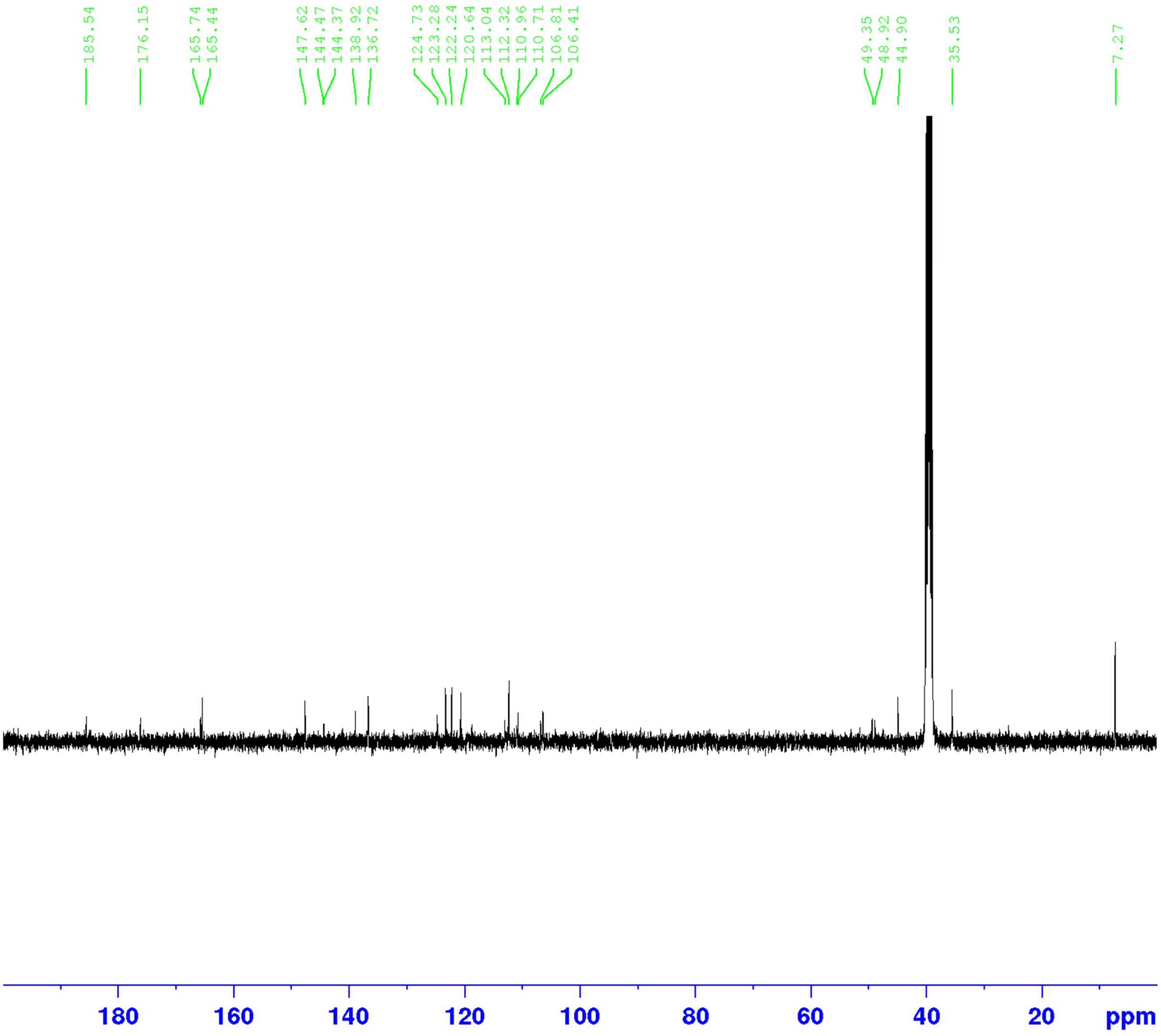
185.54
176.15
165.74
165.44
147.62
144.47
144.37
138.92
136.72
124.73
123.28
122.24
120.64
113.04
112.32
110.96
110.71
106.81
106.41
49.35
48.92
44.90
35.53
7.27
180
160
140
120
100
80
60
40
20
ppm

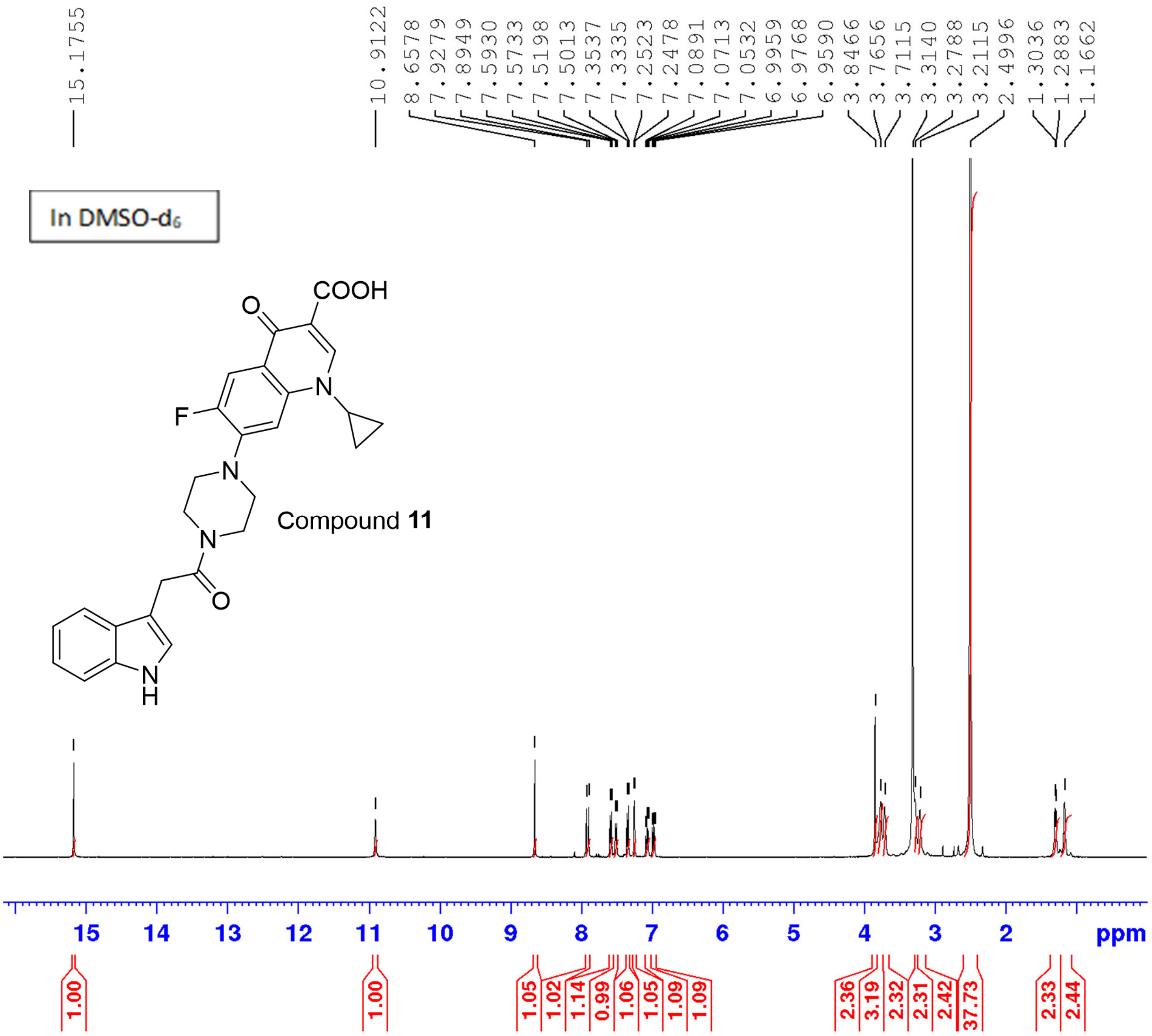
15.1755
10.9122
8.6578
7.9279
7.8949
7.5930
7.5733
7.5198
7.5013
7.3537
7.3335
7.2523
7.2478
7.0891
7.0713
7.0532
6.9959
6.9768
6.9590
3.8466
3.7656
3.7115
3.3140
3.2788
3.2115
2.4996
1.3036
1.2883
1.1662
In DMSO-d$_6$
COOH
Compound 11
15 14 13 12 11 10 9 8 7 6 5 4 3 2 ppm
1.00
1.00
1.05
1.02
1.14
0.99
1.06
1.05
1.09
1.09
2.36
3.19
2.32
2.31
2.42
37.73
2.33
2.44

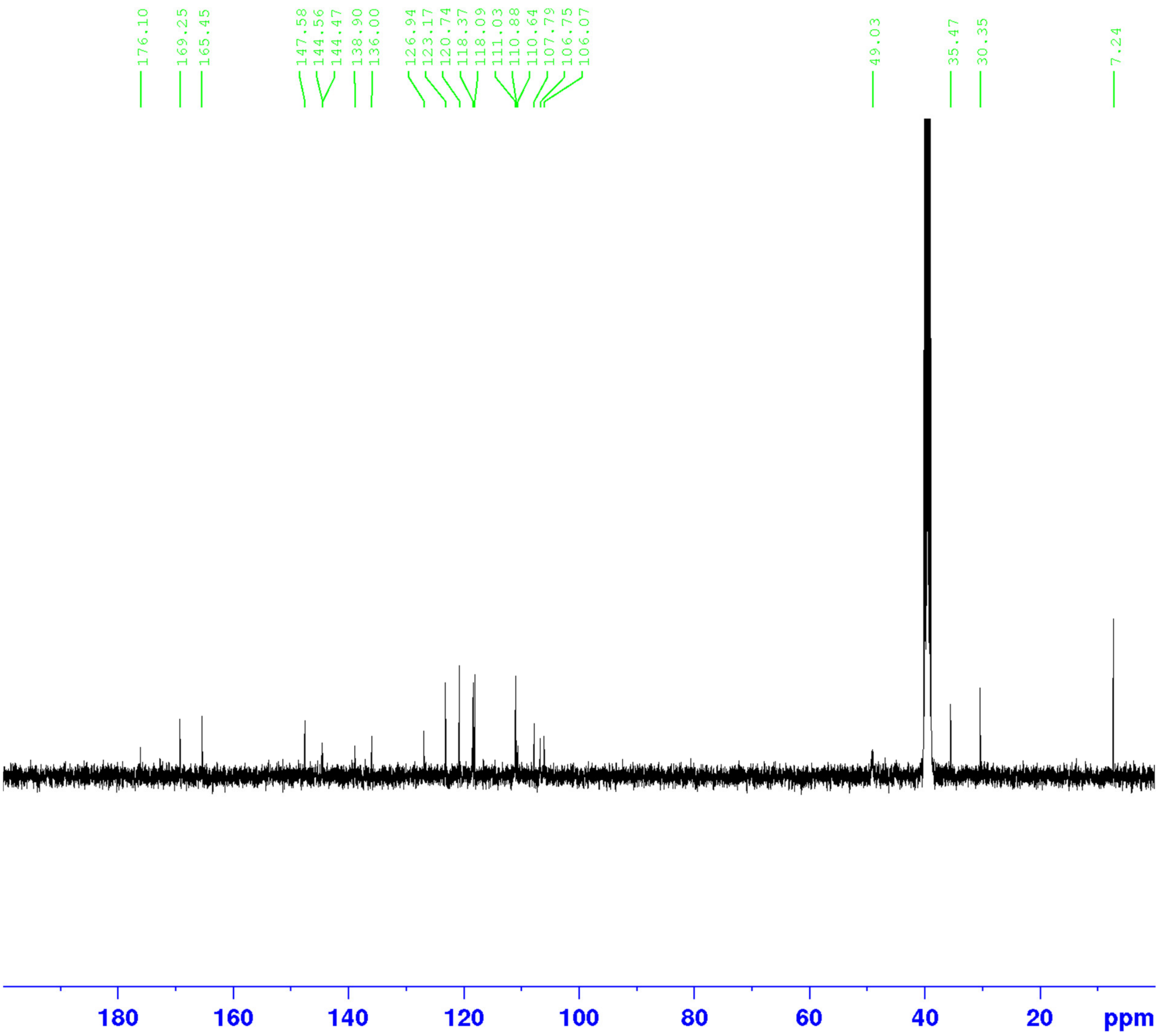
176.10
169.25
165.45
147.58
144.56
144.47
138.90
136.00
126.94
123.17
120.74
118.37
118.09
111.03
110.88
110.64
107.79
106.75
106.07
49.03
35.47
30.35
7.24
180
160
140
120
100
80
60
40
20
ppm

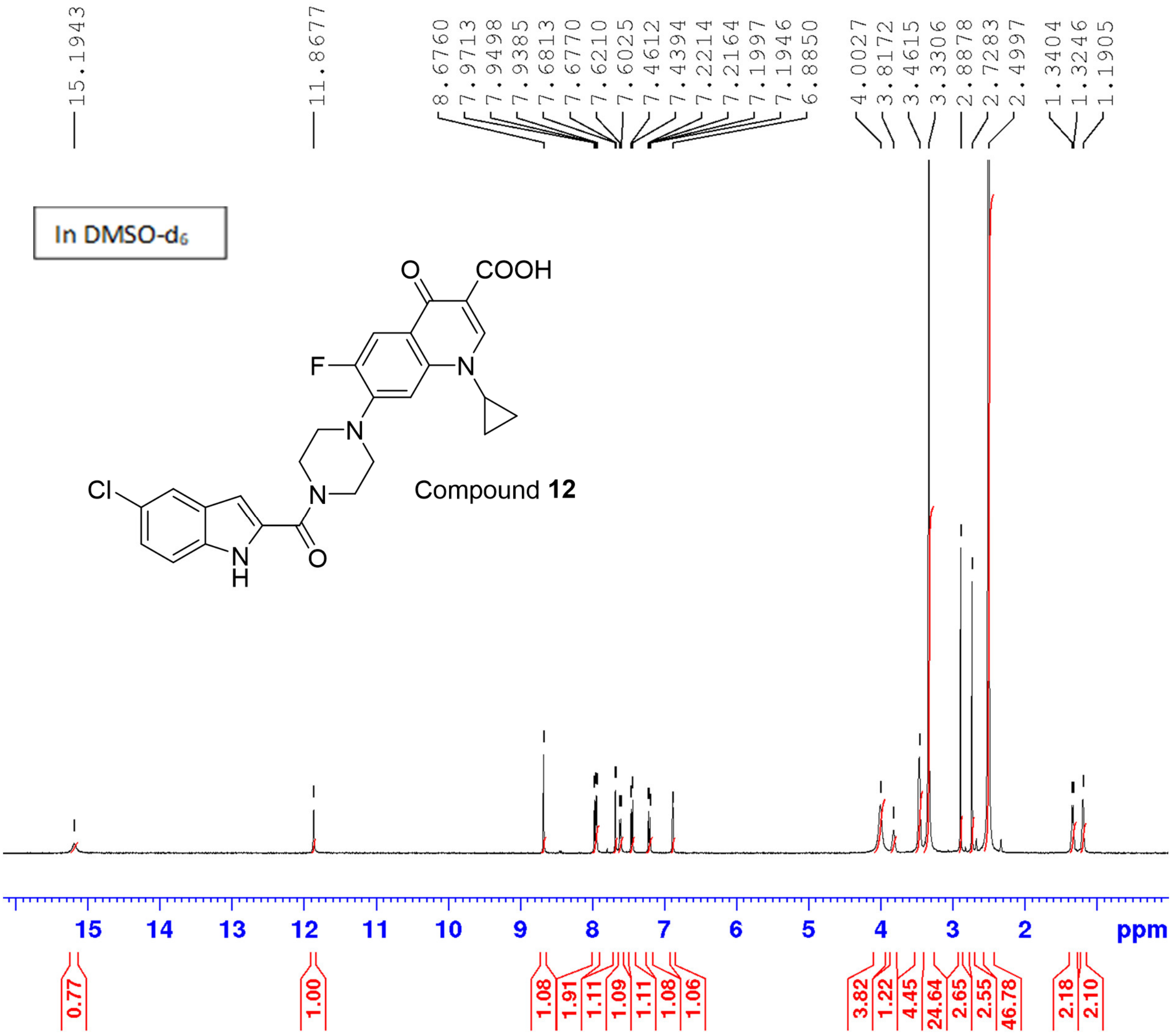
15.1943
11.8677
8.6760
7.9713
7.9498
7.9385
7.6813
7.6770
7.6210
7.6025
7.4612
7.4394
7.2214
7.2164
7.1997
7.1946
6.8850
4.0027
3.8172
3.4615
3.3306
2.8878
2.7283
2.4997
1.3404
1.3246
1.1905
In DMSO-d6
O
COOH
F
N
N
N
Cl
N
H
O
Compound 12
15 14 13 12 11 10 9 8 7 6 5 4 3 2 ppm
0.77
1.00
1.08
1.91
1.11
1.09
1.11
1.08
1.06
3.82
1.22
4.45
24.64
2.65
2.55
46.78
2.18
2.10

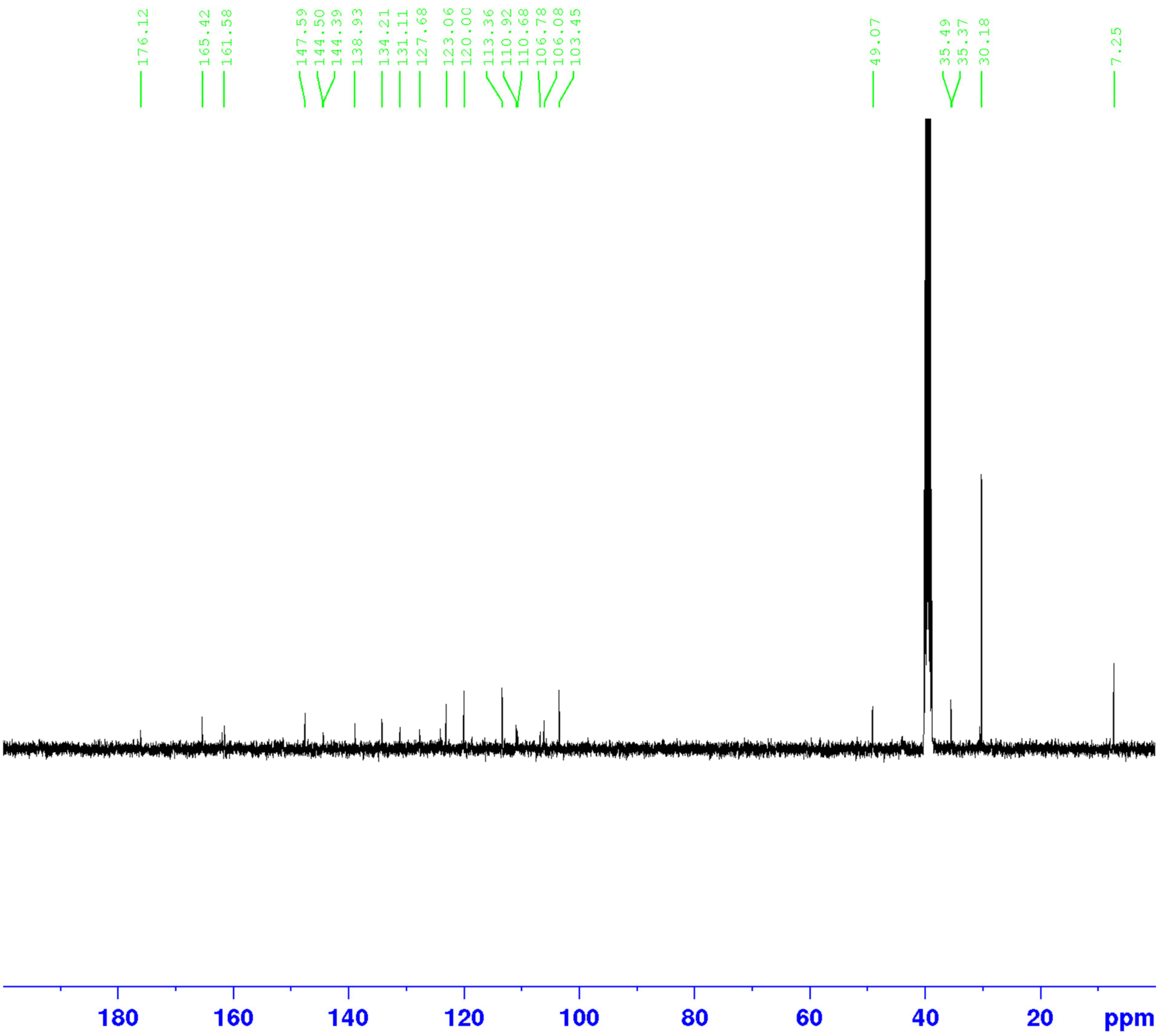
176.12
165.42
161.58
147.59
144.50
144.39
138.93
134.21
131.11
127.68
123.06
120.00
113.36
110.92
110.68
106.78
106.08
103.45
49.07
35.49
35.37
30.18
7.25
180
160
140
120
100
80
60
40
20
ppm

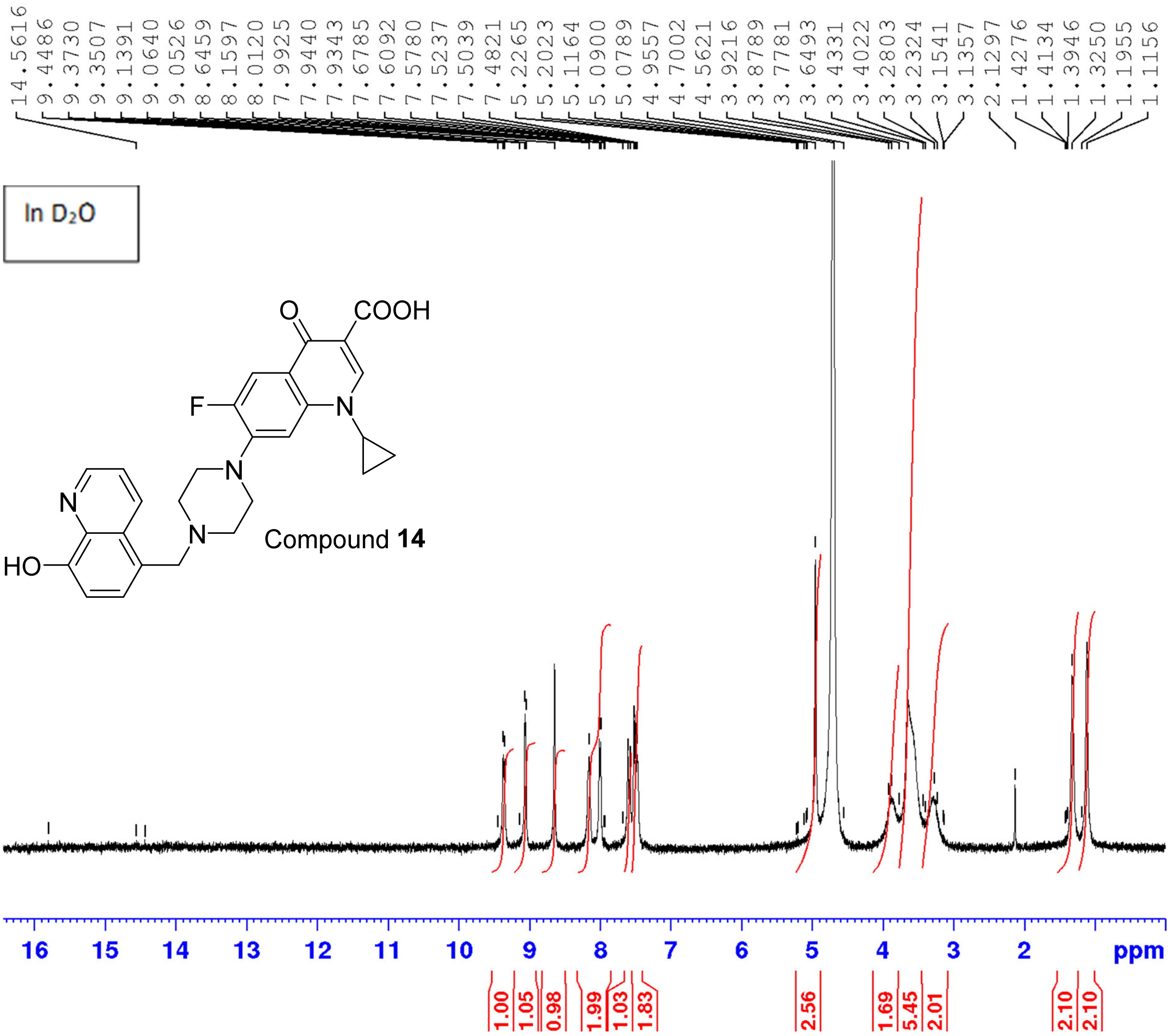
14.5616
9.4486
9.3730
9.3507
9.1391
9.0640
9.0526
8.6459
8.1597
8.0120
7.9925
7.9440
7.9343
7.6785
7.6092
7.5780
7.5237
7.5039
7.4821
5.2265
5.2023
5.1164
5.0900
5.0789
4.9557
4.7002
4.5621
3.9216
3.8789
3.7781
3.6493
3.4331
3.4022
3.2803
3.2324
3.1541
3.1357
2.1297
1.4276
1.4134
1.3946
1.3250
1.1955
1.1156
In D2O
O
COOH
F
N
N
N
N
HO
Compound 14
16 15 14 13 12 11 10 9 8 7 6 5 4 3 2 ppm
1.00
1.05
0.98
1.99
1.03
1.83
2.56
1.69
5.45
2.01
2.10
2.10

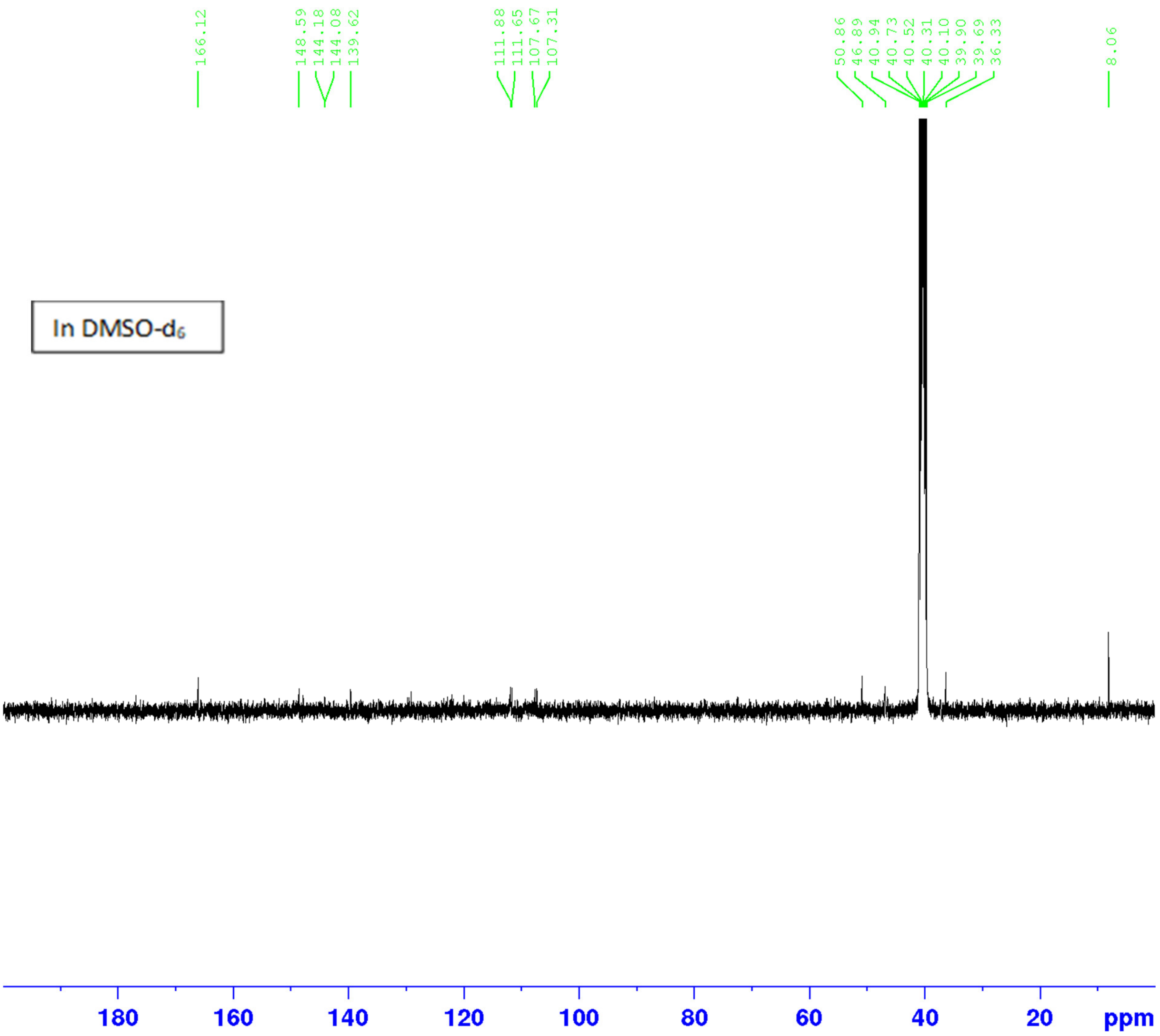
166.12
148.59
144.18
144.08
139.62
111.88
111.65
107.67
107.31
50.86
46.89
40.94
40.73
40.52
40.31
40.10
39.90
39.69
36.33
8.06
In DMSO-d6
180
160
140
120
100
80
60
40
20
ppm

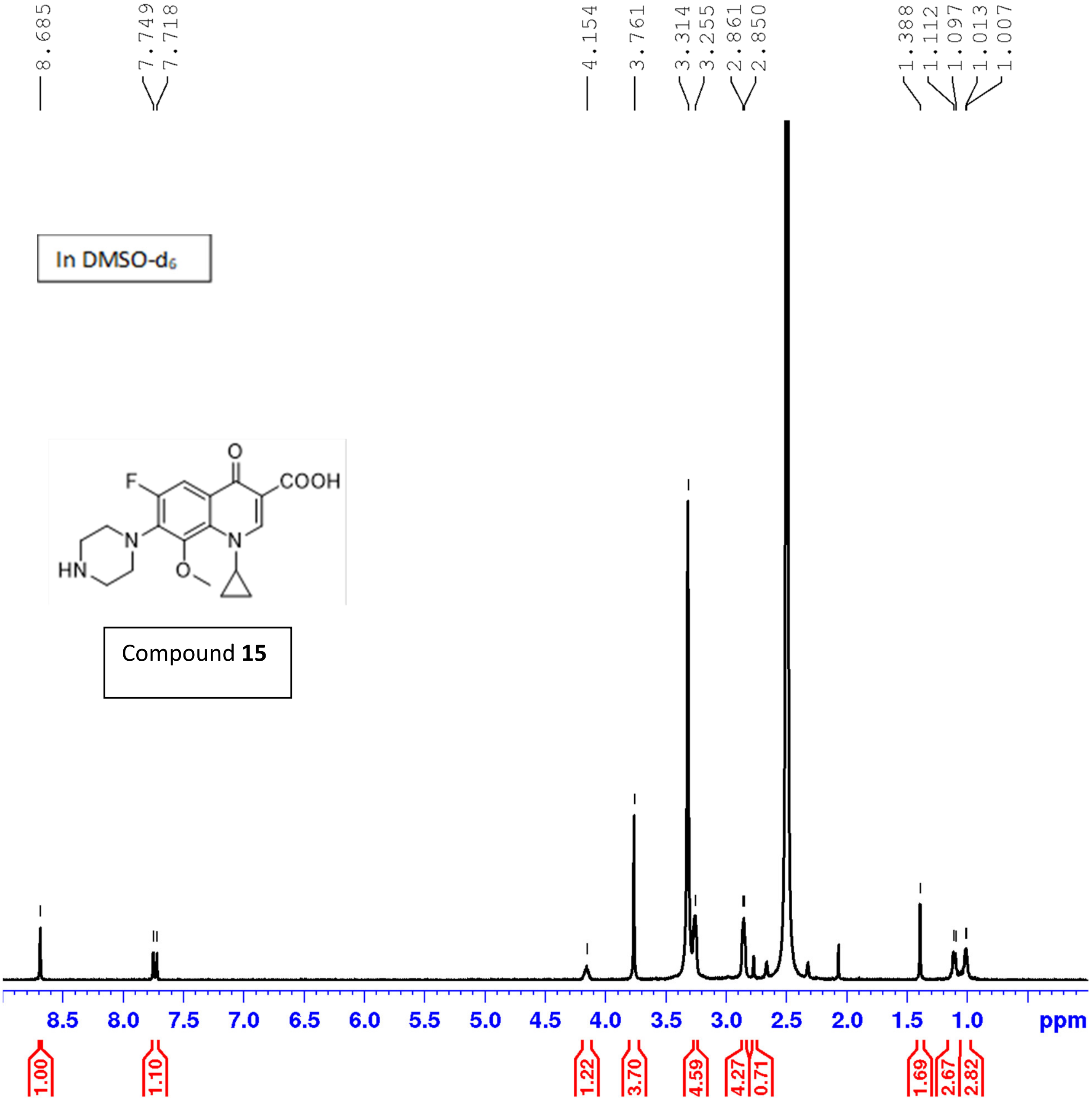
8.685
7.749
7.718
4.154
3.761
3.314
3.255
2.861
2.850
1.388
1.112
1.097
1.013
1.007
In DMSO-d6
F
O
COOH
N
N
HN
O
8.5 8.0 7.5 7.0 6.5 6.0 5.5 5.0 4.5 4.0 3.5 3.0 2.5 2.0 1.5 1.0 ppm
1.00
1.10
1.22
3.70
4.59
4.27
0.71
1.69
2.67
2.82

Compound **15**

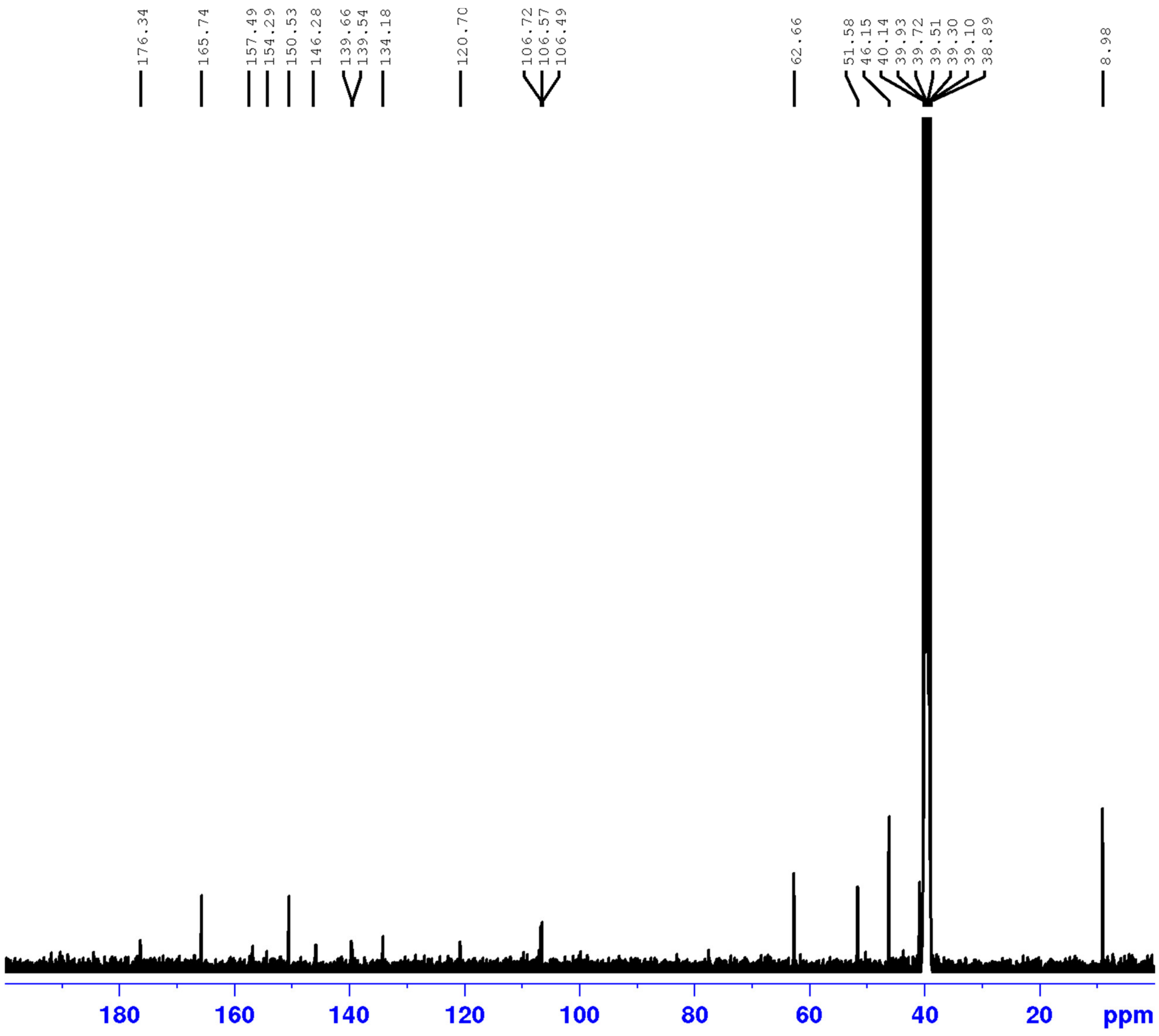
176.34
165.74
157.49
154.29
150.53
146.28
139.66
139.54
134.18
120.70
106.72
106.57
106.49
62.66
51.58
46.15
40.14
39.93
39.72
39.51
39.30
39.10
38.89
8.98
180
160
140
120
100
80
60
40
20
ppm

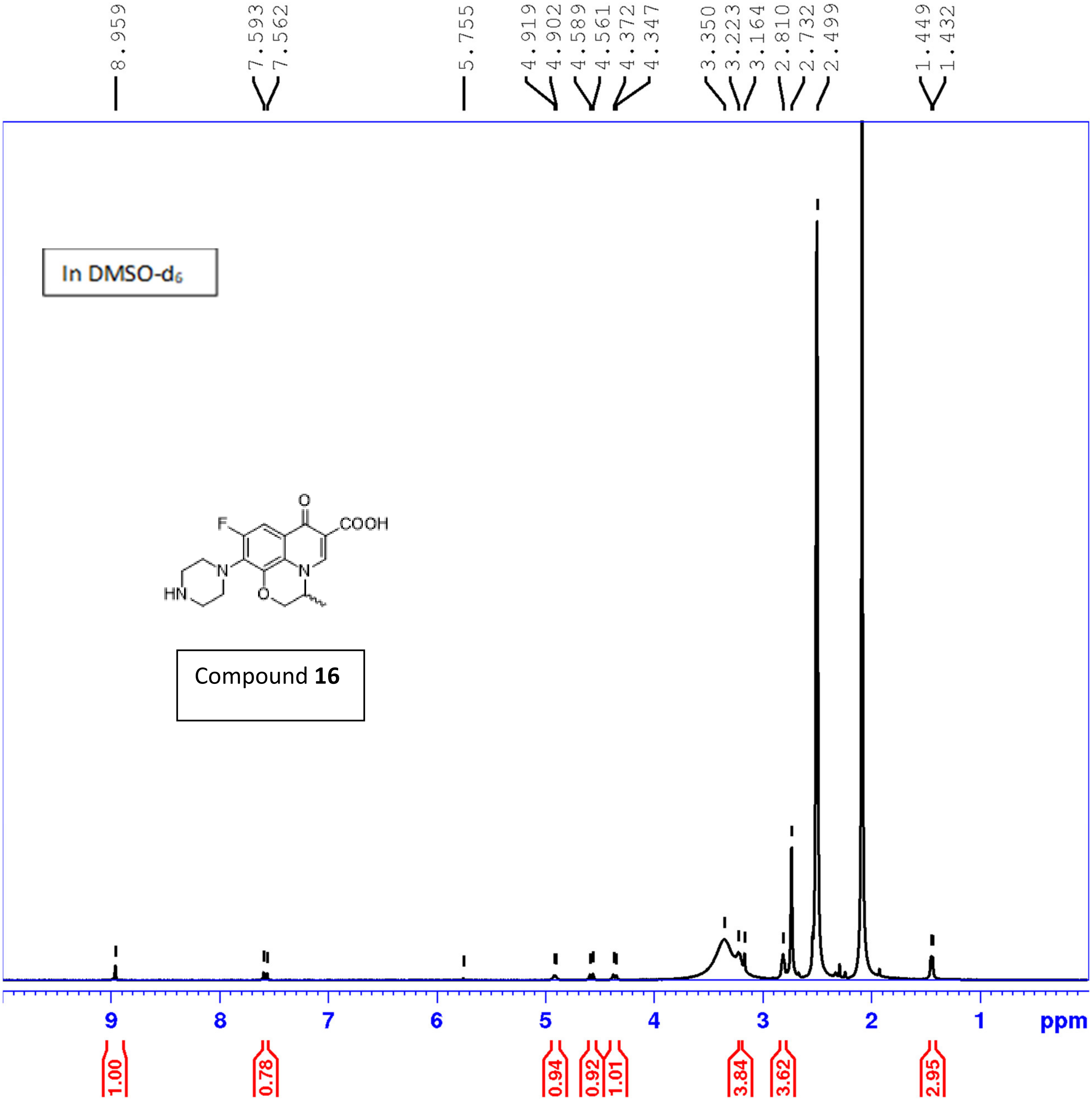
8.959
7.593
7.562
5.755
4.919
4.902
4.589
4.561
4.372
4.347
3.350
3.223
3.164
2.810
2.732
2.499
1.449
1.432
In DMSO-d6
F
COOH
O
N
HN
Compound 16
9
8
7
6
5
4
3
2
1
ppm
1.00
0.78
0.94
0.92
1.01
3.84
3.62
2.95

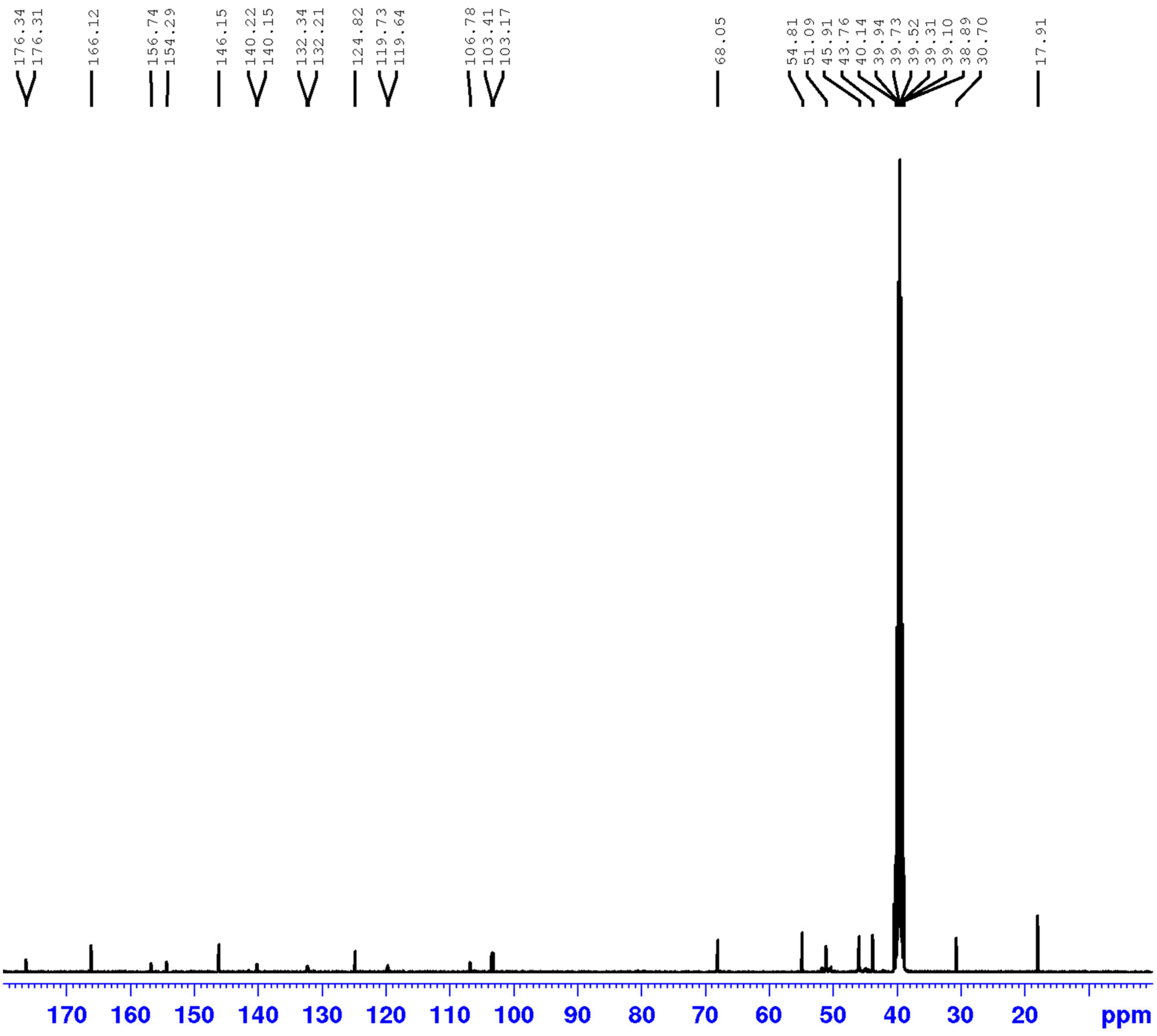
176.34
176.31
166.12
156.74
154.29
146.15
140.22
140.15
132.34
132.21
124.82
119.73
119.64
106.78
103.41
103.17
68.05
54.81
51.09
45.91
43.76
40.14
39.94
39.73
39.52
39.31
39.10
38.89
30.70
17.91
170 160 150 140 130 120 110 100 90 80 70 60 50 40 30 20 ppm

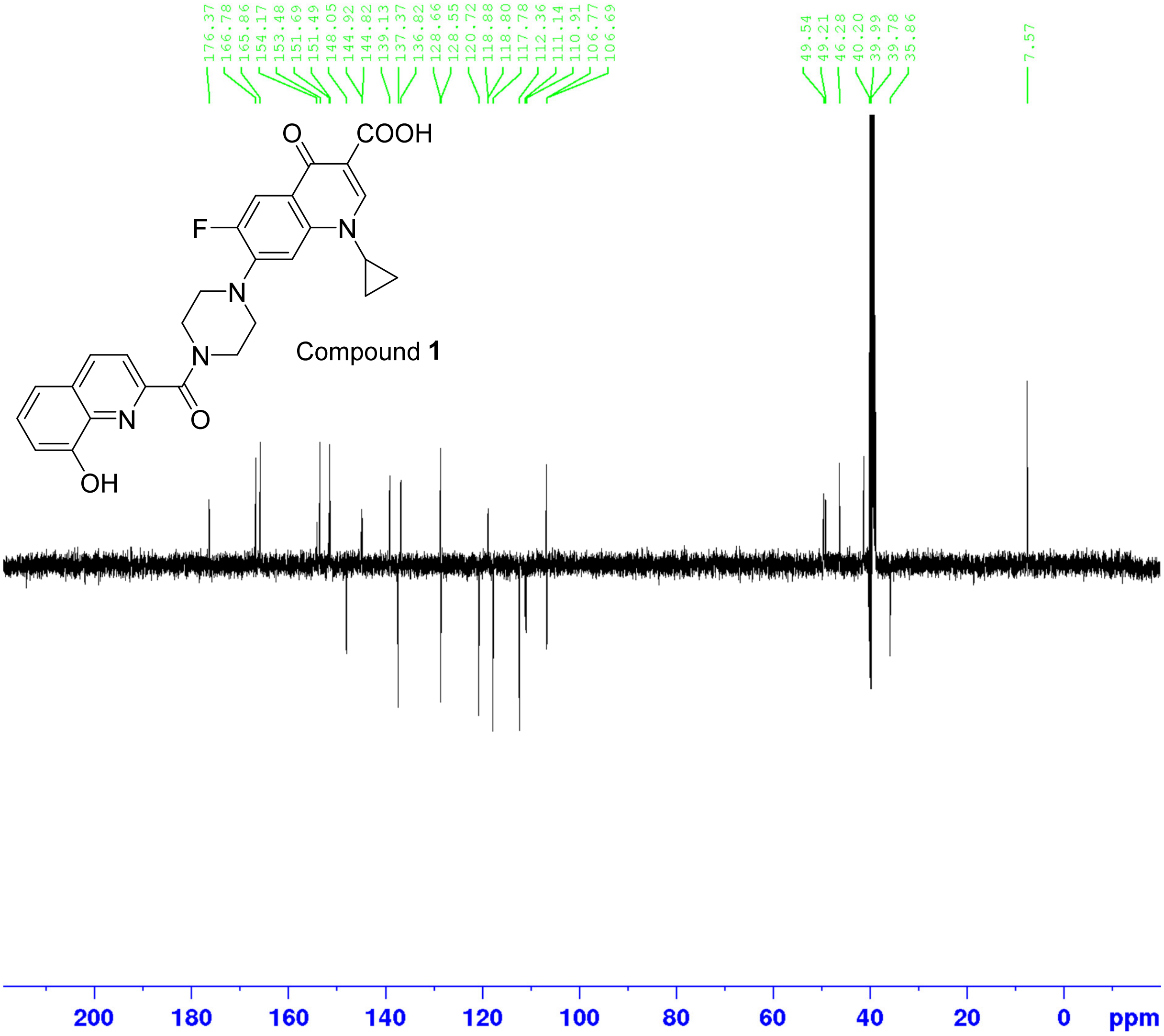
176.37
166.78
165.86
154.17
153.48
151.69
151.49
148.05
144.92
144.82
139.13
137.37
136.82
128.66
128.55
120.72
118.88
118.80
117.78
112.36
111.14
110.91
106.77
106.69
49.54
49.21
46.28
40.20
39.99
39.78
35.86
7.57
O
COOH
F
N
N
N
N
O
OH
Compound **1**
200
180
160
140
120
100
80
60
40
20
0
ppm

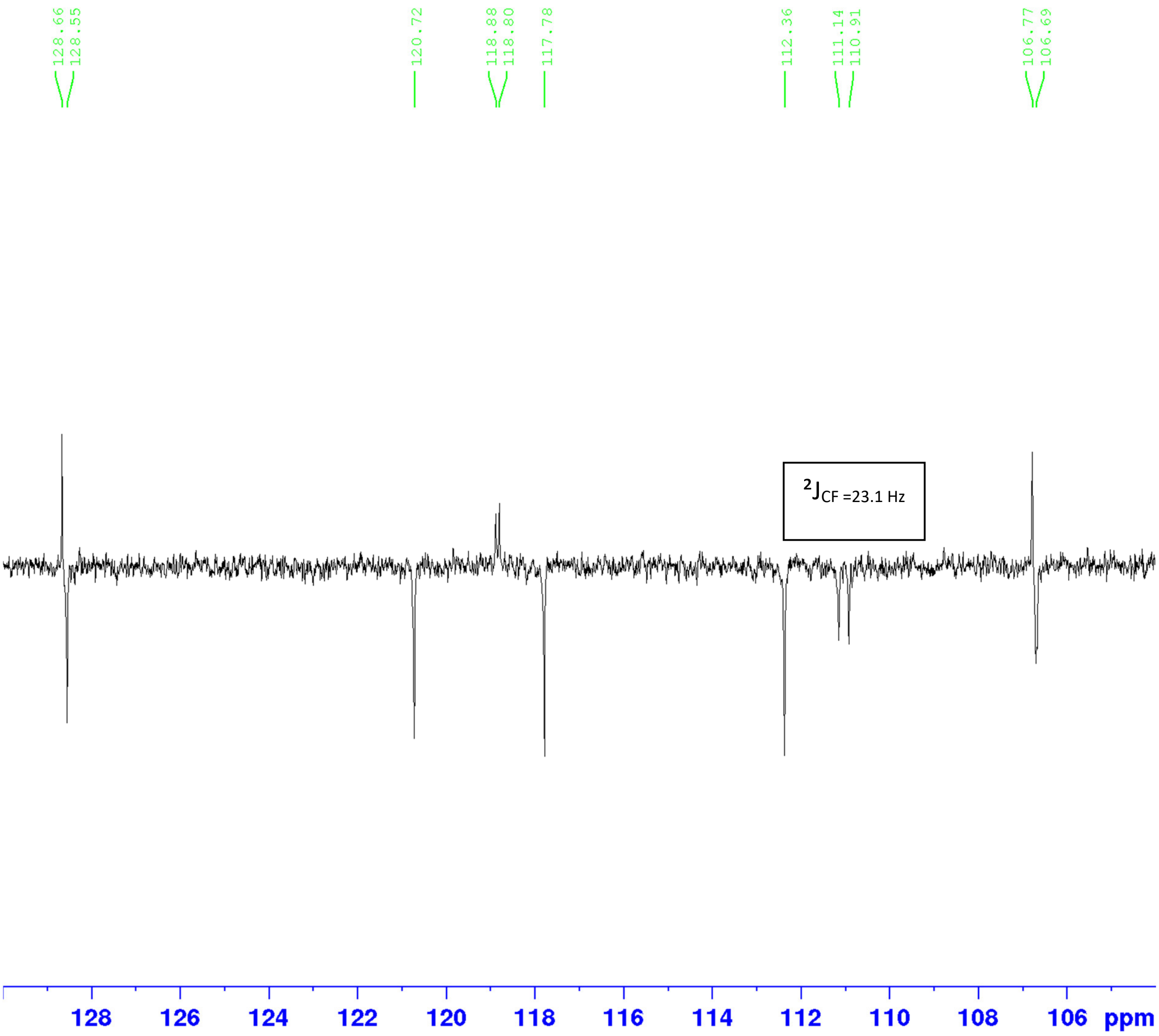
128.66
128.55
120.72
118.88
118.80
117.78
112.36
111.14
110.91
106.77
106.69
$^2J_{CF}$ =23.1 Hz
128
126
124
122
120
118
116
114
112
110
108
106
ppm

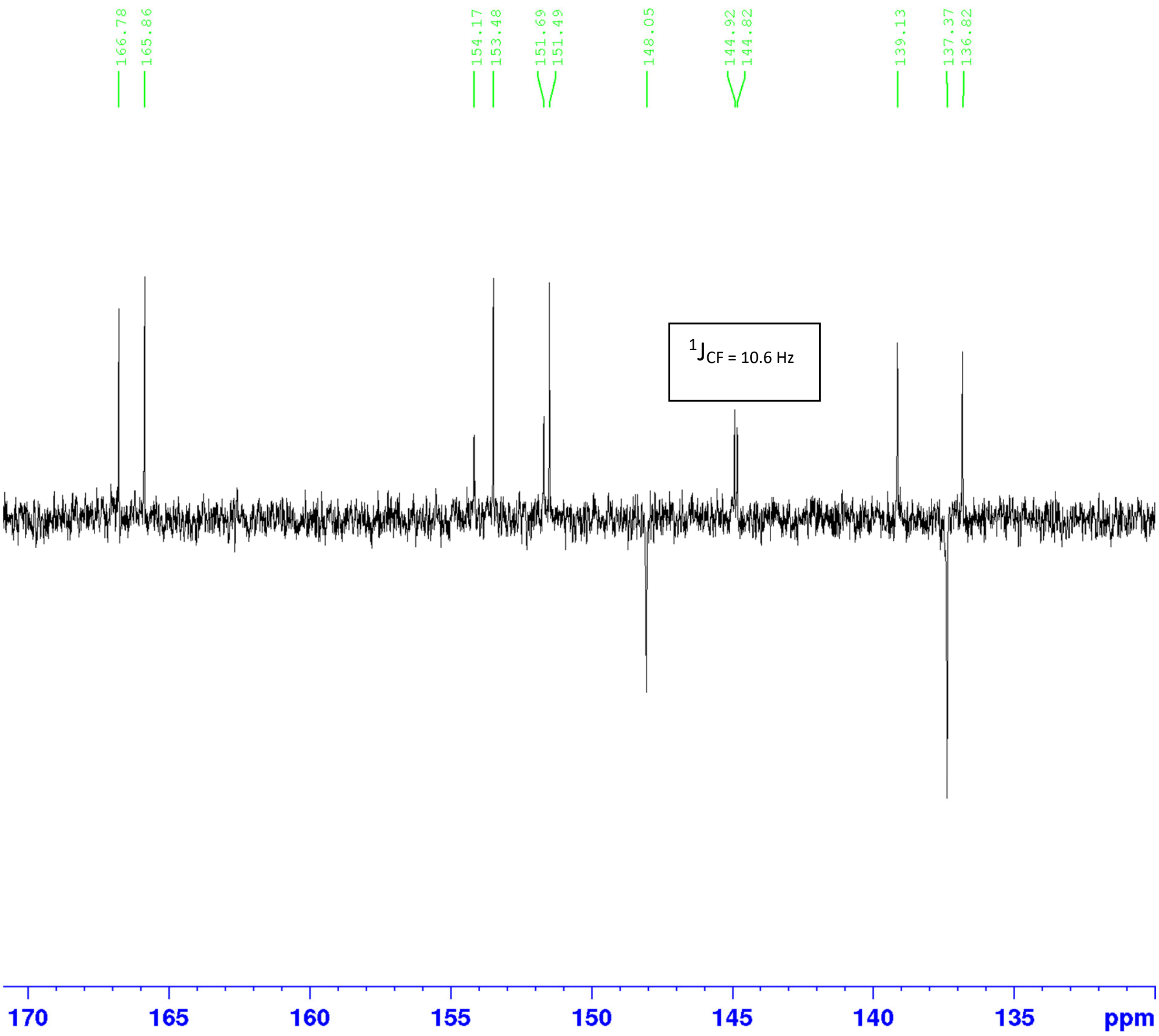

References

[1] Anquetin, G.; Greiner, J.; Mahmoudi, N.; Santillana-Hayat, M.; Gozalbes, R.; Farhati, K.; Derouin, F.; Aubry, A.; Cambau, E.; Vierling, P. Design, Synthesis and Activity against Toxoplasma Gondii, Plasmodium Spp., and Mycobacterium Tuberculosis of New 6-Fluoroquinolones. *European Journal of Medicinal Chemistry* **2006**, *41*, 1478–1493, doi:10.1016/j.ejmech.2006.07.003.
